# Supplementary material for: Serum protein N-glycome patterns reveal alterations associated with endometrial cancer and its phenotypes of differentiation
Source: Front Endocrinol (Lausanne). 2023 Jun 26;14:1157487. doi: 10.3389/fendo.2023.1157487 (PMC10331720; doi:10.3389/fendo.2023.1157487)
Supplement: Supplementary file 1 [file DataSheet_1.pdf]

Supplementary Material

Serum Protein N-glycome Patterns Reveal Alterations Associated with Endometrial Cancer and Its Phenotypes of Differentiation

Zejian Zhang*, Zhen Cao, Jinhui Wang*, Zepeng Li, Tao Wang, Yang Xiang*

*** Correspondence:** Jinhui Wang: jinhuiwang@gmail.com; Zejian Zhang: zejianzhang2018@163.com; Yang Xiang: xiangy@pumch.cn

1 Supplementary Figures and Tables

1.1 Supplementary Figures

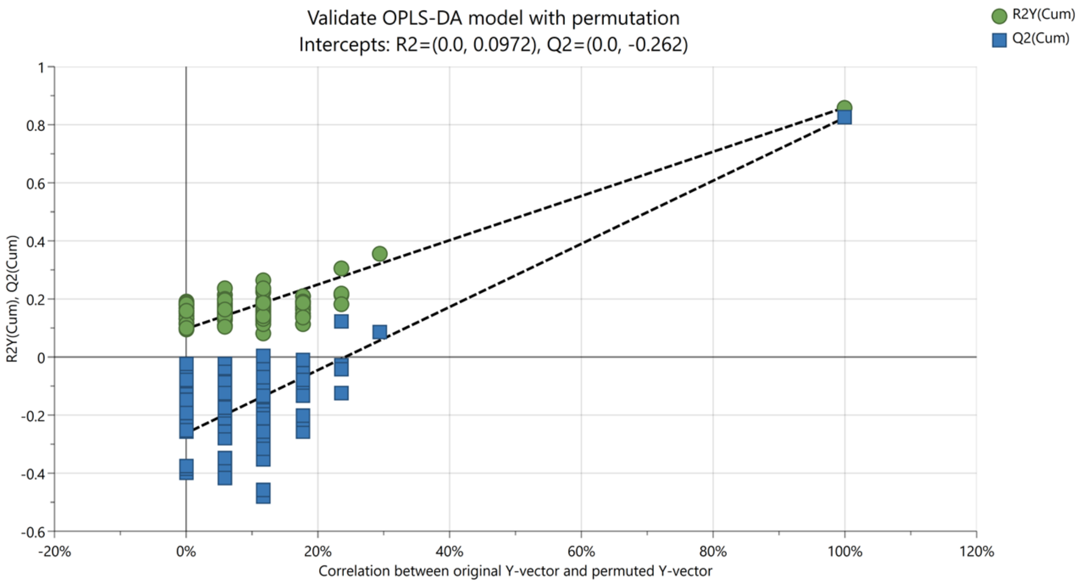

Supplementary Figure 1. The OPLS-DA model's permutation (cross-validation) test. Permutation number = 200. OPLS-DA, orthogonal partial least squares - discriminant analysis.

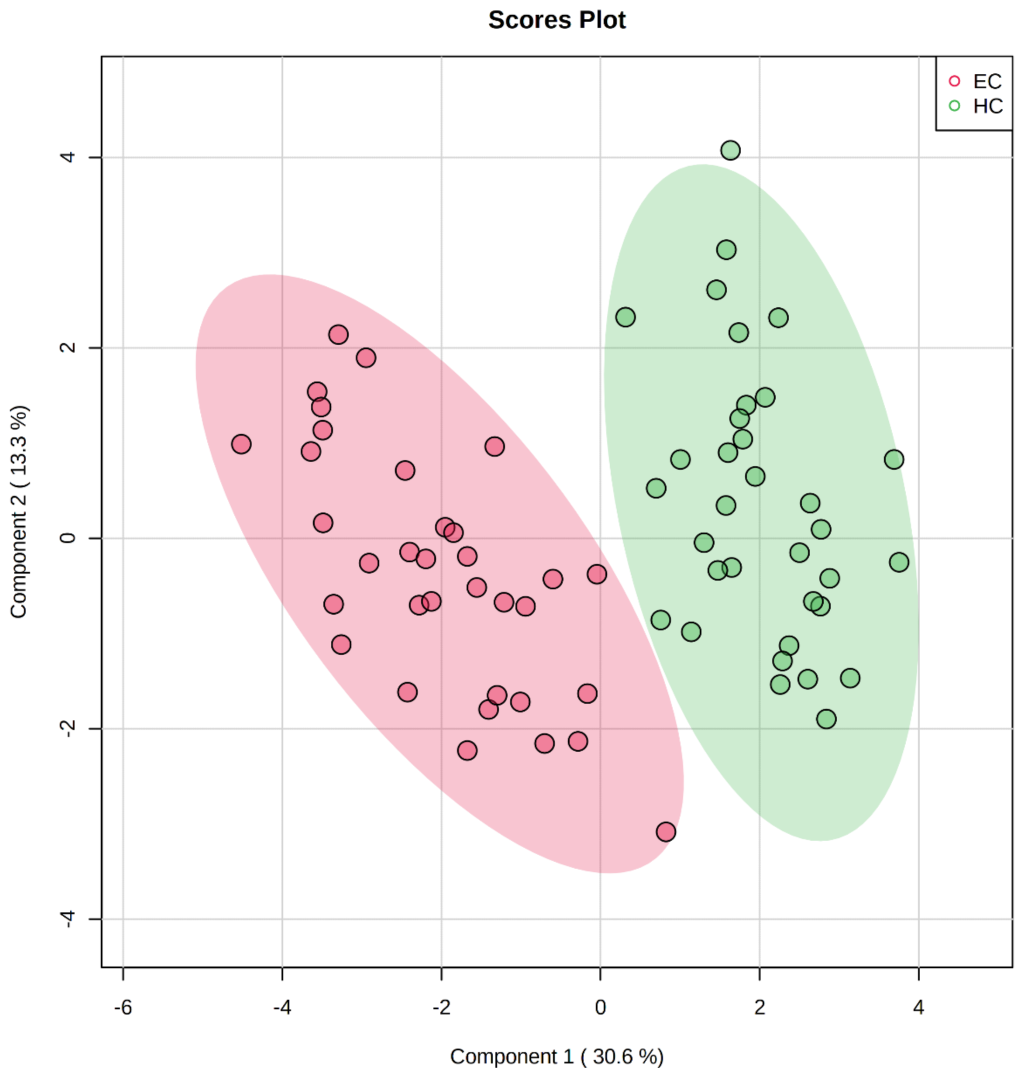

Supplementary Figure 2. Scores plot of the sparse partial least squares - discriminant analysis (sPLS-DA) between endometrial cancer (EC, red circle) and healthy controls (HC, green circle) groups. The scores plot is based on derived glycan traits. Areas of 95% confidence interval are highlighted in red and green for EC and HC, respectively.

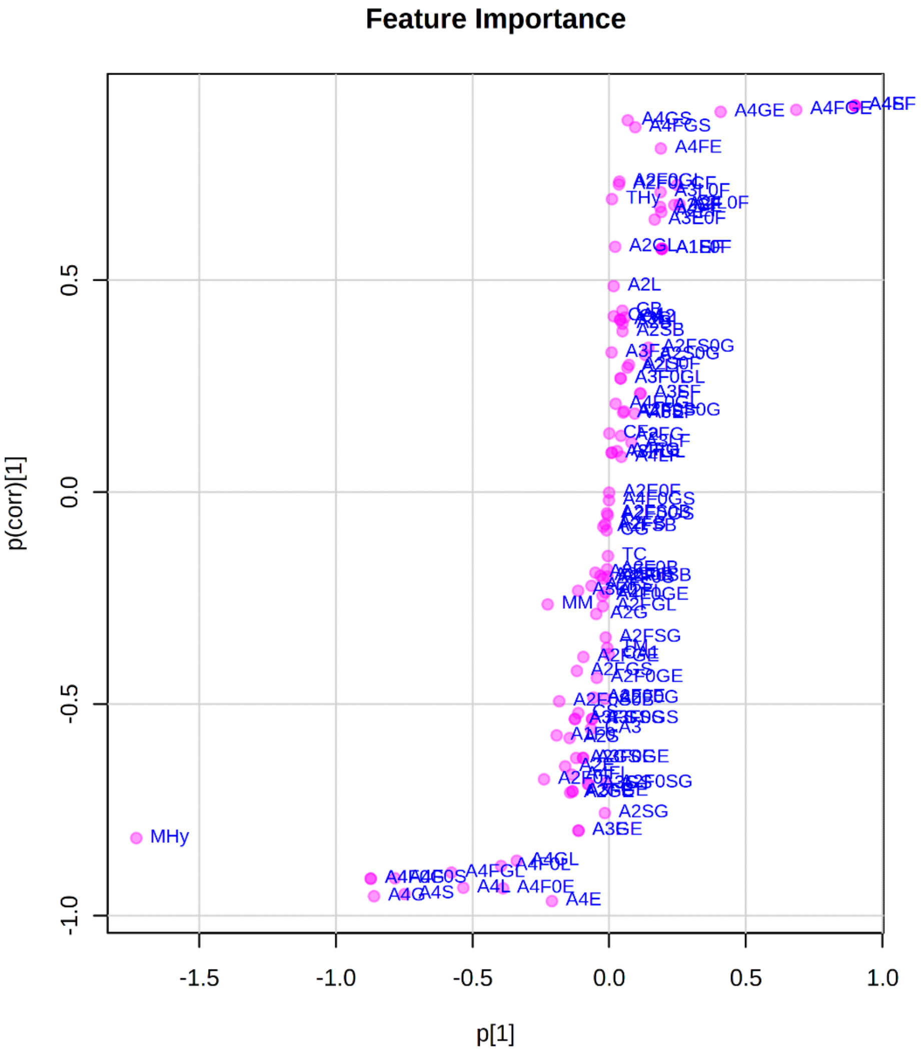

Supplementary Figure 3. The S-plot of the orthogonal partial least squares - discriminant analysis (OPLS-DA).

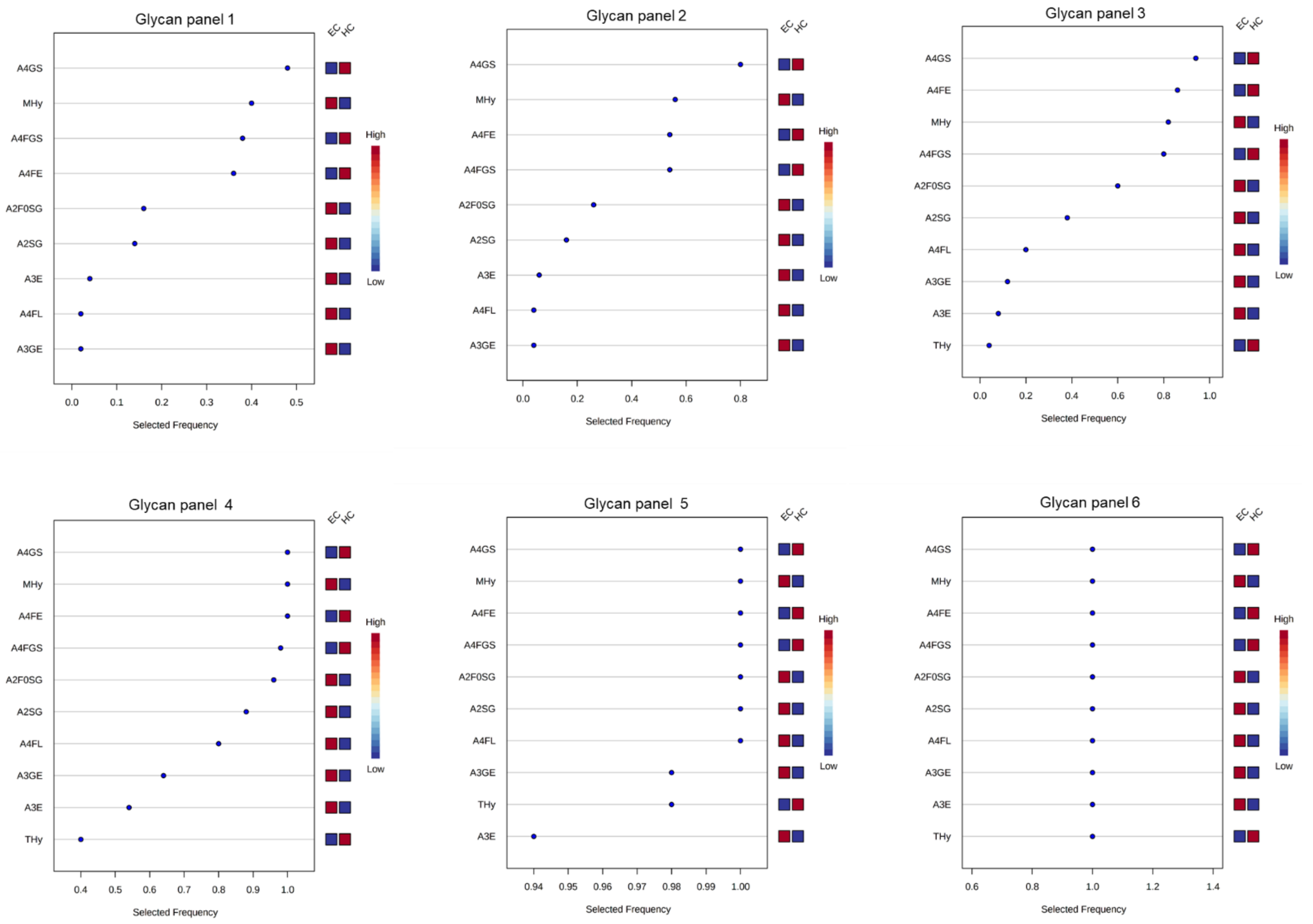

Supplementary Figure 4. The most frequently selected derived glycan traits when building the glycan panels (panel 1-panel 6, built with 2, 3, 5, 10, 20, or 33 derived glycan traits). They were selected and evaluated by the multivariate receiver operating characteristic (ROC) exploratory analyses. The exploratory analysis didn’t point out which specific derived glycan traits were used to construct each glycan panel, but it indicated the most frequently selected derived glycan traits when building the panels, which facilitate the building of the optimal glycan panel.

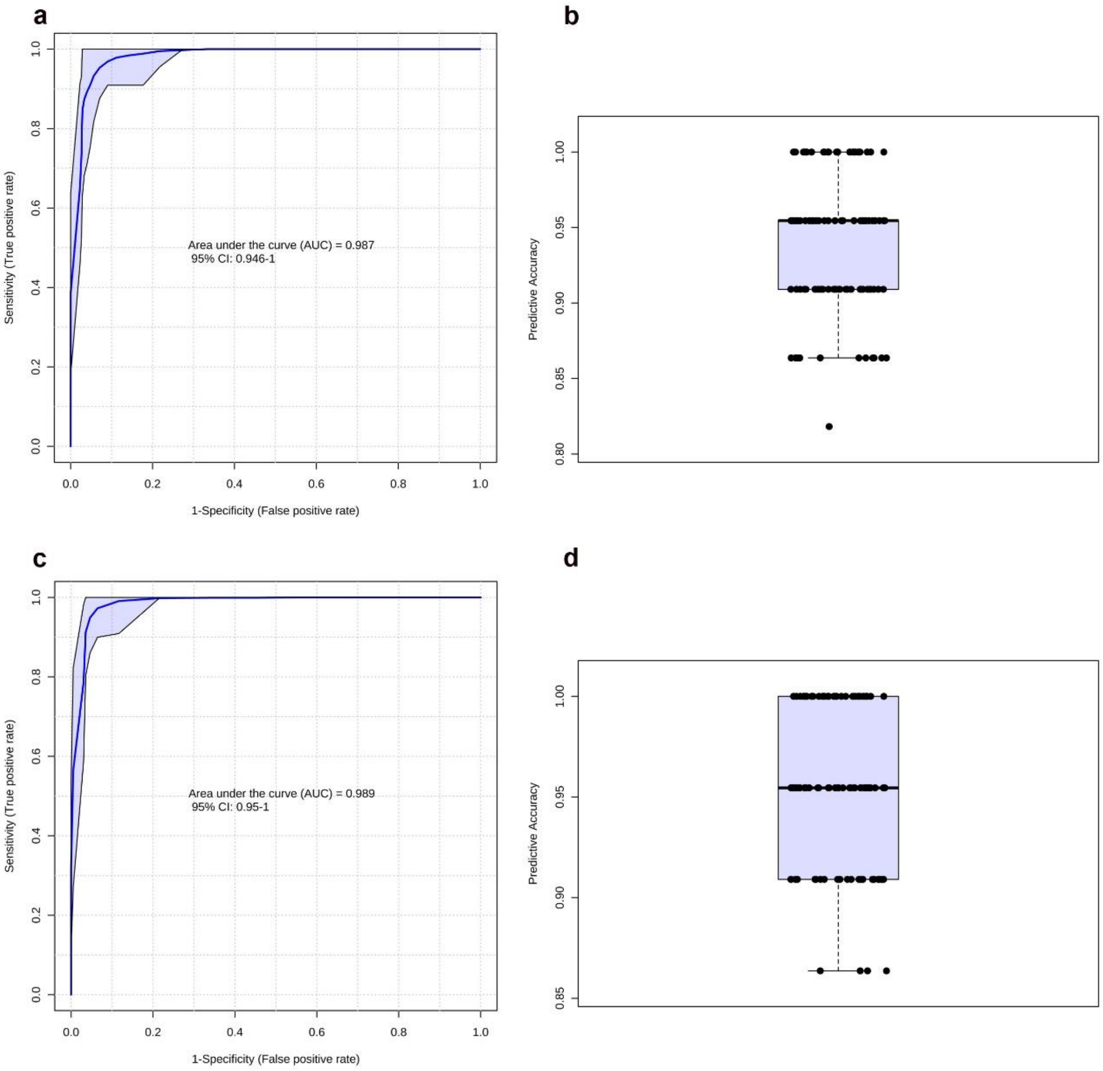

Supplementary Figure 5. Diagnostic performance of the optimized glycan panel built with the four most important (discriminative and biologically reliable) glycan traits based on the support vector machines (SVM) model/algorithm and partial least squares (PLS) model/algorithm for endometrial cancer. (A) receiver operating characteristic (ROC) curve and (B) the predictive accuracy based on the linear SVM model/algorithm. (C) ROC curve and (D) the predictive accuracy based on the PLS model/algorithm.

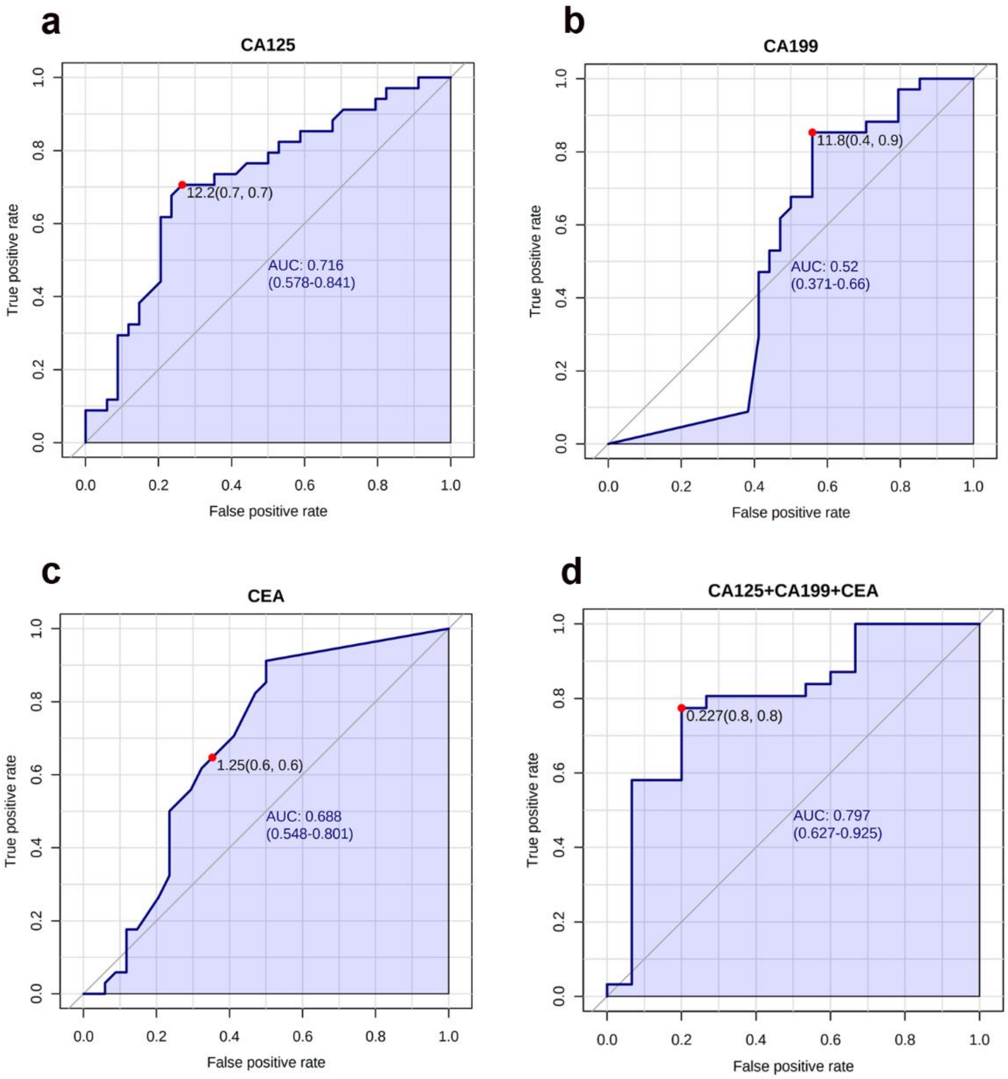

Supplementary Figure 6. Diagnostic performance of the classical gynecologic tumor markers for endometrial cancer. The diagnostic performance of (A) carbohydrate antigen 125 [CA125], (B) CA199, (C) carcinoembryonic antigen [CEA], and (D) the combination of the three markers.

1.2 Supplementary Tables

Supplementary Table 1. Overview of the directly detected N-glycans used for analysis in the present study. Compositions were detected by MALDI-TOF-MS. The glycans were released from serum samples and the N-acetylneuraminicacids of the glycans were derivatized by ethyl esterification. All species are assigned $[M+Na]^+$. H = hexose; N = N-acetylhexosamine; F = deoxyhexose (fucose); L = lactonized N-acetylneuraminic acid (α2,3-linked); E = ethyl esterified N-acetylneuraminic acid (α2,6-linked).

**Observed**	**Input composition**					**Modifiers**			**Calculated**		**Error**		**Used for calibration (marked with "*")**
Mass	H	N	F	L	E	H_2O	Na+	Ac	Composition	Mass	error (Da)	error (ppm)	
m/z	162.0528234	203.0793725	146.0579088	273.0848518	319.1267166	18.01056468	22.9892207	42.01056		*m/z*			
1257.42921	5	2	0	0	0	1	1	0	H5N2	1257.42265	0.007	5.22	
1282.44957	3	3	1	0	0	1	1	0	H3N3F1	1282.45428	-0.005	-3.68	
1339.46207	3	4	0	0	0	1	1	0	H3N4	1339.47575	-0.014	-10.21	
1419.46371	6	2	0	0	0	1	1	0	H6N2	1419.47547	-0.012	-8.28	
1444.51274	4	3	1	0	0	1	1	0	H4N3F1	1444.50711	0.006	3.90	
1455.51697	3	3	0	0	1	1	1	0	H3N3E1	1455.52309	-0.006	-4.20	
1460.52117	5	3	0	0	0	1	1	0	H5N3	1460.50202	0.019	13.11	
1485.54111	3	4	1	0	0	1	1	0	H3N4F1	1485.53365	0.007	5.02	*
1501.53349	4	4	0	0	0	1	1	0	H4N4	1501.52857	0.005	3.28	
1542.54968	3	5	0	0	0	1	1	0	H3N5	1542.55512	-0.005	-3.52	
1581.52308	7	2	0	0	0	1	1	0	H7N2	1581.52829	-0.005	-3.29	
1601.59564	3	3	1	0	1	1	1	0	H3N3F1E1	1601.58100	0.015	9.14	
1606.55678	5	3	1	0	0	1	1	0	H5N3F1	1606.55993	-0.003	-1.96	

1617.58387	4	3	0	0	1	1	1	0	H4N3E1	1617.57591	0.008	4.92	
1622.55001	6	3	0	0	0	1	1	0	H6N3	1622.55484	-0.005	-2.98	
1647.59562	4	4	1	0	0	1	1	0	H4N4F1	1647.58648	0.009	5.55	*
1663.59579	5	4	0	0	0	1	1	0	H5N4	1663.58139	0.014	8.65	
1688.61932	3	5	1	0	0	1	1	0	H3N5F1	1688.61303	0.006	3.73	
1704.60044	4	5	0	0	0	1	1	0	H4N5	1704.60794	-0.008	-4.40	
1733.58716	5	3	0	1	0	1	1	0	H5N3L1	1733.58687	0.000	0.17	
1743.57833	8	2	0	0	0	1	1	0	H8N2	1743.58112	-0.003	-1.60	
1763.62662	4	3	1	0	1	1	1	0	H4N3F1E1	1763.63382	-0.007	-4.08	
1774.61358	4	4	0	1	0	1	1	0	H4N4L1	1774.61342	0.000	0.09	
1779.63464	5	3	0	0	1	1	1	0	H5N3E1	1779.62874	0.006	3.32	
1809.64000	5	4	1	0	0	1	1	0	H5N4F1	1809.63930	0.001	0.38	*
1820.64791	4	4	0	0	1	1	1	0	H4N4E1	1820.65529	-0.007	-4.05	
1825.60996	6	4	0	0	0	1	1	0	H6N4	1825.63422	-0.024	-13.28	
1850.66616	4	5	1	0	0	1	1	0	H4N5F1	1850.66585	0.000	0.17	
1866.65007	5	5	0	0	0	1	1	0	H5N5	1866.66077	-0.011	-5.73	
1905.63145	9	2	0	0	0	1	1	0	H9N2	1905.63394	-0.002	-1.31	
1936.65929	5	4	0	1	0	1	1	0	H5N4L1	1936.66624	-0.007	-3.59	
1941.67229	6	3	0	0	1	1	1	0	H6N3E1	1941.68156	-0.009	-4.77	
1966.72426	4	4	1	0	1	1	1	0	H4N4F1E1	1966.71319	0.011	5.62	
1982.71002	5	4	0	0	1	1	1	0	H5N4E1	1982.70811	0.002	0.96	*
2012.72088	5	5	1	0	0	1	1	0	H5N5F1	2012.71867	0.002	1.09	
2023.74760	4	5	0	0	1	1	1	0	H4N5E1	2023.73466	0.013	6.40	
2082.74927	5	4	1	1	0	1	1	0	H5N4F1L1	2082.72415	0.025	12.06	

2098.71588	6	4	0	1	0	1	1	0	H6N4L1	2098.71907	-0.003	-1.52	
2110.77031	4	7	0	0	0	1	1	0	H4N7	2110.76669	0.004	1.72	
2128.77245	5	4	1	0	1	1	1	0	H5N4F1E1	2128.76602	0.006	3.02	*
2169.81455	4	5	1	0	1	1	1	0	H4N5F1E1	2169.79257	0.022	10.13	
2185.79705	5	5	0	0	1	1	1	0	H5N5E1	2185.78748	0.010	4.38	
2209.75876	5	4	0	2	0	1	1	0	H5N4L2	2209.75110	0.008	3.47	
2255.79246	5	4	0	1	1	1	1	0	H5N4E1L1	2255.79296	0.000	-0.22	
2301.83999	5	4	0	0	2	1	1	0	H5N4E2	2301.83483	0.005	2.25	
2331.85508	5	5	1	0	1	1	1	0	H5N5F1E1	2331.84539	0.010	4.15	
2347.84336	6	5	0	0	1	1	1	0	H6N5E1	2347.84031	0.003	1.30	
2355.78851	5	4	1	2	0	1	1	0	H5N4F1L2	2355.80900	-0.020	-8.70	
2372.86451	4	6	1	0	1	1	1	0	H4N6F1E1	2372.87194	-0.007	-3.13	
2401.85567	5	4	1	1	1	1	1	0	H5N4F1E1L1	2401.85087	0.005	2.00	
2429.90998	4	7	0	0	1	1	1	0	H4N7E1	2429.89340	0.017	6.82	
2447.89465	5	4	1	0	2	1	1	0	H5N4F1E2	2447.89273	0.002	0.78	
2493.89248	6	5	1	0	1	1	1	0	H6N5F1E1	2493.89821	-0.006	-2.30	
2504.91878	5	5	0	0	2	1	1	0	H5N5E2	2504.91420	0.005	1.83	
2574.89939	6	5	0	2	0	1	1	0	H6N5L2	2574.88329	0.016	6.25	
2604.92781	5	5	1	1	1	1	1	0	H5N5F1E1L1	2604.93024	-0.002	-0.93	
2620.91774	6	5	0	1	1	1	1	0	H6N5E1L1	2620.92516	-0.007	-2.83	
2650.96604	5	5	1	0	2	1	1	0	H5N5F1E2	2650.97211	-0.006	-2.29	
2666.94791	6	5	0	0	2	1	1	0	H6N5E2	2666.96702	-0.019	-7.16	
2720.91983	6	5	1	2	0	1	1	0	H6N5F1L2	2720.94120	-0.021	-7.85	
2766.98562	6	5	1	1	1	1	1	0	H6N5F1E1L1	2766.98307	0.003	0.92	

2813.00526	6	5	1	0	2	1	1	0	H6N5F1E2	2813.02493	-0.020	-6.99	
2848.00774	8	6	2	0	0	1	1	0	H8N6F2	2848.01443	-0.007	-2.35	
2894.02407	6	5	0	2	1	1	1	0	H6N5E1L2	2894.01001	0.014	4.86	
2940.04523	6	5	0	1	2	1	1	0	H6N5E2L1	2940.05187	-0.007	-2.26	*
2986.06421	6	5	0	0	3	1	1	0	H6N5E3	2986.09374	-0.030	-9.89	
3040.04173	6	5	1	2	1	1	1	0	H6N5F1E1L2	3040.06792	-0.026	-8.61	
3086.11533	6	5	1	1	2	1	1	0	H6N5F1E2L1	3086.10978	0.006	1.80	*
3132.13278	6	5	1	0	3	1	1	0	H6N5F1E3	3132.15165	-0.019	-6.02	
3232.18571	6	5	2	1	2	1	1	0	H6N5F2E2L1	3232.16769	0.018	5.58	
3259.13248	7	6	0	2	1	1	1	0	H7N6E1L2	3259.14220	-0.010	-2.98	
3305.20626	7	6	0	1	2	1	1	0	H7N6E2L1	3305.18407	0.022	6.71	
3405.16982	7	6	1	2	1	1	1	0	H7N6F1E1L2	3405.20011	-0.030	-8.90	
3451.24806	7	6	1	1	2	1	1	0	H7N6F1E2L1	3451.24198	0.006	1.76	
3532.21170	7	6	0	3	1	1	1	0	H7N6E1L3	3532.22706	-0.015	-4.35	*
3578.25147	7	6	0	2	2	1	1	0	H7N6E2L2	3578.26892	-0.017	-4.88	
3624.30007	7	6	0	1	3	1	1	0	H7N6E3L1	3624.31079	-0.011	-2.96	
3678.30979	7	6	1	3	1	1	1	0	H7N6F1E1L3	3678.28497	0.025	6.75	
3724.32403	7	6	1	2	2	1	1	0	H7N6F1E2L2	3724.32683	-0.003	-0.75	

Supplementary Table 2. Description, depiction, and calculation of derived glycan traits. M = mannose; Hy = hybrid species; T = within total spectrum; C = within complex species; F = deoxyhexose (fucose); G = galactose; S = N-acetylneuraminic acid (sialic acid); E = α2,6-linked sialic acid; L = α2,3-linked sialic acid; H = hexose (mannose or galactose); N = N-acetylhexosamine (N-acetylglucosamine: GlcNAc).

**Derived traits**	**Description**	**Formular of calculation (derived glycan traits were calculated from the directly detected glycans)**
**Glycan type**		
**TM**	Relative abundance of high mannose type glycans within total spectrum	TM = (H5N2 + H6N2 + H7N2 + H8N2 + H9N2) / (H5N2 + H3N3F1 + H3N4 + H6N2 + H4N3F1 + H3N3E1 + H5N3 + H3N4F1 + H4N4 + H3N5 + H7N2 + H3N3F1E1 + H5N3F1 + H4N3E1 + H6N3 + H4N4F1 + H5N4 + H3N5F1 + H4N5 + H5N3L1 + H8N2 + H4N3F1E1 + H4N4L1 + H5N3E1 + H5N4F1 + H4N4E1 + H6N4 + H4N5F1 + H5N5 + H9N2 + H5N4L1 + H6N3E1 + H4N4F1E1 + H5N4E1 + H5N5F1 + H4N5E1 + H5N4F1L1 + H6N4L1 + H4N7 + H5N4F1E1 + H4N5F1E1 + H5N5E1 + H5N4L2 + H5N4E1L1 + H5N4E2 + H5N5F1E1 + H6N5E1 + H5N4F1L2 + H4N6F1E1 + H5N4F1E1L1 + H4N7E1 + H5N4F1E2 + H6N5F1E1 + H5N5E2 + H6N5L2 + H5N5F1E1L1 + H6N5E1L1 + H5N5F1E2 + H6N5E2 + H6N5F1L2 + H6N5F1E1L1 + H6N5F1E2 + H8N6F2 + H6N5E1L2 + H6N5E2L1 + H6N5E3 + H6N5F1E1L2 + H6N5F1E2L1 + H6N5F1E3 + H6N5F2E2L1 + H7N6E1L2 + H7N6E2L1 + H7N6F1E1L2 + H7N6F1E2L1 + H7N6E1L3 + H7N6E2L2 + H7N6E3L1 + H7N6F1E1L3 + H7N6F1E2L2)
**THy**	Relative abundance of hybrid type glycans within total spectrum	THy = (H5N3 + H5N3F1 + H6N3 + H5N3L1 + H5N3E1 + H6N4 + H6N3E1 + H6N4L1 + H8N6F2) / (H5N2 + H3N3F1 + H3N4 + H6N2 + H4N3F1 + H3N3E1 + H5N3 + H3N4F1 + H4N4 + H3N5 + H7N2 + H3N3F1E1 + H5N3F1 + H4N3E1 + H6N3 + H4N4F1 + H5N4 + H3N5F1 + H4N5 + H5N3L1 + H8N2 + H4N3F1E1 + H4N4L1 + H5N3E1 + H5N4F1 + H4N4E1 + H6N4 + H4N5F1 + H5N5 + H9N2 + H5N4L1 + H6N3E1 + H4N4F1E1 + H5N4E1 + H5N5F1 + H4N5E1 + H5N4F1L1 + H6N4L1 + H4N7 + H5N4F1E1 + H4N5F1E1 + H5N5E1 + H5N4L2 + H5N4E1L1 + H5N4E2 + H5N5F1E1 + H6N5E1 + H5N4F1L2 + H4N6F1E1 + H5N4F1E1L1 + H4N7E1 + H5N4F1E2 + H6N5F1E1 + H5N5E2 + H6N5L2 + H5N5F1E1L1 + H6N5E1L1 + H5N5F1E2 + H6N5E2 + H6N5F1L2 + H6N5F1E1L1 + H6N5F1E2 + H8N6F2 + H6N5E1L2 + H6N5E2L1 + H6N5E3 + H6N5F1E1L2 + H6N5F1E2L1 + H6N5F1E3 + H6N5F2E2L1 + H7N6E1L2 + H7N6E2L1 + H7N6F1E1L2 + H7N6F1E2L1 + H7N6E1L3 + H7N6E2L2 + H7N6E3L1 + H7N6F1E1L3 + H7N6F1E2L2)

**TC**	Total complex glycans	TC = (H3N4 + H3N3E1 + H3N4F1 + H4N4 + H3N5 + H3N3F1E1 + H4N3E1 + H4N4F1 + H5N4 + H3N5F1 + H4N5 + H4N3F1E1 + H4N4L1 + H5N4F1 + H4N4E1 + H4N5F1 + H5N5 + H5N4L1 + H4N4F1E1 + H5N4E1 + H5N5F1 + H4N5E1 + H5N4F1L1 + H4N7 + H5N4F1E1 + H4N5F1E1 + H5N5E1 + H5N4L2 + H5N4E1L1 + H5N4E2 + H5N5F1E1 + H6N5E1 + H5N4F1L2 + H4N6F1E1 + H5N4F1E1L1 + H4N7E1 + H5N4F1E2 + H6N5F1E1 + H5N5E2 + H6N5L2 + H5N5F1E1L1 + H6N5E1L1 + H5N5F1E2 + H6N5E2 + H6N5F1L2 + H6N5F1E1L1 + H6N5F1E2 + H6N5E1L2 + H6N5E2L1 + H6N5E3 + H6N5F1E1L2 + H6N5F1E2L1 + H6N5F1E3 + H6N5F2E2L1 + H7N6E1L2 + H7N6E2L1 + H7N6F1E1L2 + H7N6F1E2L1 + H7N6E1L3 + H7N6E2L2 + H7N6E3L1 + H7N6F1E1L3 + H7N6F1E2L2) / (H5N2 + H3N3F1 + H3N4 + H6N2 + H4N3F1 + H3N3E1 + H5N3 + H3N4F1 + H4N4 + H3N5 + H7N2 + H3N3F1E1 + H5N3F1 + H4N3E1 + H6N3 + H4N4F1 + H5N4 + H3N5F1 + H4N5 + H5N3L1 + H8N2 + H4N3F1E1 + H4N4L1 + H5N3E1 + H5N4F1 + H4N4E1 + H6N4 + H4N5F1 + H5N5 + H9N2 + H5N4L1 + H6N3E1 + H4N4F1E1 + H5N4E1 + H5N5F1 + H4N5E1 + H5N4F1L1 + H6N4L1 + H4N7 + H5N4F1E1 + H4N5F1E1 + H5N5E1 + H5N4L2 + H5N4E1L1 + H5N4E2 + H5N5F1E1 + H6N5E1 + H5N4F1L2 + H4N6F1E1 + H5N4F1E1L1 + H4N7E1 + H5N4F1E2 + H6N5F1E1 + H5N5E2 + H6N5L2 + H5N5F1E1L1 + H6N5E1L1 + H5N5F1E2 + H6N5E2 + H6N5F1L2 + H6N5F1E1L1 + H6N5F1E2 + H8N6F2 + H6N5E1L2 + H6N5E2L1 + H6N5E3 + H6N5F1E1L2 + H6N5F1E2L1 + H6N5F1E3 + H6N5F2E2L1 + H7N6E1L2 + H7N6E2L1 + H7N6F1E1L2 + H7N6F1E2L1 + H7N6E1L3 + H7N6E2L2 + H7N6E3L1 + H7N6F1E1L3 + H7N6F1E2L2)
**MHy**	The ratio of high-mannose to hybrid glycans	MHy = (H5N2 + H6N2 + H7N2 + H8N2 + H9N2) / (H5N3 + H5N3F1 + H6N3 + H5N3L1 + H5N3E1 + H6N4 + H6N3E1 + H6N4L1 + H8N6F2)
**MM**	Average number of mannoses on high mannose type glycans	MM = (5 * (H5N2) + 6 * (H6N2) + 7 * (H7N2) + 8 * (H8N2) + 9 * (H9N2) + 10 * (0)) / (H5N2 + H6N2 + H7N2 + H8N2 + H9N2)
**CA1**	Relative abundance of monoantennary glycans within complex type glycans	CA1 = (H3N3E1 + H3N3F1E1 + H4N3E1 + H4N3F1E1) / (H3N4 + H3N3E1 + H3N4F1 + H4N4 + H3N5 + H3N3F1E1 + H4N3E1 + H4N4F1 + H5N4 + H3N5F1 + H4N5 + H4N3F1E1 + H4N4L1 + H5N4F1 + H4N4E1 + H4N5F1 + H5N5 + H5N4L1 + H4N4F1E1 + H5N4E1 + H5N5F1 + H4N5E1 + H5N4F1L1 + H4N7 + H5N4F1E1 + H4N5F1E1 + H5N5E1 + H5N4L2 + H5N4E1L1 + H5N4E2 + H5N5F1E1 + H6N5E1 + H5N4F1L2 + H4N6F1E1 + H5N4F1E1L1 + H4N7E1 + H5N4F1E2 + H6N5F1E1 + H5N5E2 + H6N5L2 + H5N5F1E1L1 + H6N5E1L1 + H5N5F1E2 + H6N5E2 + H6N5F1L2 + H6N5F1E1L1 + H6N5F1E2 + H6N5E1L2 + H6N5E2L1 + H6N5E3 + H6N5F1E1L2 + H6N5F1E2L1 + H6N5F1E3 + H6N5F2E2L1 + H7N6E1L2 + H7N6E2L1 + H7N6F1E1L2 + H7N6F1E2L1 + H7N6E1L3 + H7N6E2L2 + H7N6E3L1 + H7N6F1E1L3 + H7N6F1E2L2)
**CA2**	Relative abundance of diantennary glycans within complex type glycans	CA2 = (H3N4 + H3N4F1 + H4N4 + H3N5 + H4N4F1 + H5N4 + H3N5F1 + H4N5 + H4N4L1 + H5N4F1 + H4N4E1 + H4N5F1 + H5N5 + H5N4L1 + H4N4F1E1 + H5N4E1 + H5N5F1 + H4N5E1 + H5N4F1L1 + H5N4F1E1 + H4N5F1E1 + H5N5E1 + H5N4L2 + H5N4E1L1 + H5N4E2 + H5N5F1E1 + H5N4F1L2 + H5N4F1E1L1 + H5N4F1E2 + H5N5E2 + H5N5F1E1L1 + H5N5F1E2) / (H3N4 + H3N3E1 + H3N4F1 + H4N4 + H3N5 + H3N3F1E1 + H4N3E1 + H4N4F1 + H5N4 + H3N5F1 + H4N5 + H4N3F1E1 + H4N4L1 + H5N4F1 + H4N4E1 + H4N5F1 + H5N5 + H5N4L1 + H4N4F1E1 + H5N4E1 + H5N5F1 + H4N5E1 + H5N4F1L1 + H4N7 + H5N4F1E1 + H4N5F1E1 + H5N5E1 + H5N4L2 + H5N4E1L1 + H5N4E2 + H5N5F1E1 + H6N5E1 + H5N4F1L2 + H4N6F1E1 + H5N4F1E1L1 + H4N7E1 + H5N4F1E2 + H6N5F1E1 + H5N5E2 + H6N5L2 + H5N5F1E1L1 + H6N5E1L1 + H5N5F1E2 + H6N5E2 + H6N5F1L2 + H6N5F1E1L1 + H6N5F1E2 + H6N5E1L2 + H6N5E2L1 + H6N5E3 + H6N5F1E1L2 + H6N5F1E2L1 + H6N5F1E3 + H6N5F2E2L1 + H7N6E1L2 + H7N6E2L1 + H7N6F1E1L2 + H7N6F1E2L1 + H7N6E1L3 + H7N6E2L2 + H7N6E3L1 + H7N6F1E1L3 + H7N6F1E2L2)

**CA3**	Relative abundance of triantennary glycans within complex type glycans	CA3 = (H6N5E1 + H6N5F1E1 + H6N5L2 + H6N5E1L1 + H6N5E2 + H6N5F1L2 + H6N5F1E1L1 + H6N5F1E2 + H6N5E1L2 + H6N5E2L1 + H6N5E3 + H6N5F1E1L2 + H6N5F1E2L1 + H6N5F1E3 + H6N5F2E2L1) / (H3N4 + H3N3E1 + H3N4F1 + H4N4 + H3N5 + H3N3F1E1 + H4N3E1 + H4N4F1 + H5N4 + H3N5F1 + H4N5 + H4N3F1E1 + H4N4L1 + H5N4F1 + H4N4E1 + H4N5F1 + H5N5 + H5N4L1 + H4N4F1E1 + H5N4E1 + H5N5F1 + H4N5E1 + H5N4F1L1 + H4N7 + H5N4F1E1 + H4N5F1E1 + H5N5E1 + H5N4L2 + H5N4E1L1 + H5N4E2 + H5N5F1E1 + H6N5E1 + H5N4F1L2 + H4N6F1E1 + H5N4F1E1L1 + H4N7E1 + H5N4F1E2 + H6N5F1E1 + H5N5E2 + H6N5L2 + H5N5F1E1L1 + H6N5E1L1 + H5N5F1E2 + H6N5E2 + H6N5F1L2 + H6N5F1E1L1 + H6N5F1E2 + H6N5E1L2 + H6N5E2L1 + H6N5E3 + H6N5F1E1L2 + H6N5F1E2L1 + H6N5F1E3 + H6N5F2E2L1 + H7N6E1L2 + H7N6E2L1 + H7N6F1E1L2 + H7N6F1E2L1 + H7N6E1L3 + H7N6E2L2 + H7N6E3L1 + H7N6F1E1L3 + H7N6F1E2L2)
**CA4**	Relative abundance of tetra-antennary glycans within complex type glycans	CA4 = (H4N6F1E1 + H7N6E1L2 + H7N6E2L1 + H7N6F1E1L2 + H7N6F1E2L1 + H7N6E1L3 + H7N6E2L2 + H7N6E3L1 + H7N6F1E1L3 + H7N6F1E2L2) / (H3N4 + H3N3E1 + H3N4F1 + H4N4 + H3N5 + H3N3F1E1 + H4N3E1 + H4N4F1 + H5N4 + H3N5F1 + H4N5 + H4N3F1E1 + H4N4L1 + H5N4F1 + H4N4E1 + H4N5F1 + H5N5 + H5N4L1 + H4N4F1E1 + H5N4E1 + H5N5F1 + H4N5E1 + H5N4F1L1 + H4N7 + H5N4F1E1 + H4N5F1E1 + H5N5E1 + H5N4L2 + H5N4E1L1 + H5N4E2 + H5N5F1E1 + H6N5E1 + H5N4F1L2 + H4N6F1E1 + H5N4F1E1L1 + H4N7E1 + H5N4F1E2 + H6N5F1E1 + H5N5E2 + H6N5L2 + H5N5F1E1L1 + H6N5E1L1 + H5N5F1E2 + H6N5E2 + H6N5F1L2 + H6N5F1E1L1 + H6N5F1E2 + H6N5E1L2 + H6N5E2L1 + H6N5E3 + H6N5F1E1L2 + H6N5F1E2L1 + H6N5F1E3 + H6N5F2E2L1 + H7N6E1L2 + H7N6E2L1 + H7N6F1E1L2 + H7N6F1E2L1 + H7N6E1L3 + H7N6E2L2 + H7N6E3L1 + H7N6F1E1L3 + H7N6F1E2L2)
**TA2FS0 (IgG specific glycans)**	Total asialo fucosylated A2	TA2FS0 = (H3N4F1 + H4N4F1 + H3N5F1 + H5N4F1 + H4N5F1 + H5N5F1)
**Fucosylation**		
**CF**	Fucosylation within complex type glycans	CF = (H3N4F1 + H3N3F1E1 + H4N4F1 + H3N5F1 + H4N3F1E1 + H5N4F1 + H4N5F1 + H4N4F1E1 + H5N5F1 + H5N4F1L1 + H5N4F1E1 + H4N5F1E1 + H5N5F1E1 + H5N4F1L2 + H4N6F1E1 + H5N4F1E1L1 + H5N4F1E2 + H6N5F1E1 + H5N5F1E1L1 + H5N5F1E2 + H6N5F1L2 + H6N5F1E1L1 + H6N5F1E2 + H6N5F1E1L2 + H6N5F1E2L1 + H6N5F1E3 + H6N5F2E2L1 + H7N6F1E1L2 + H7N6F1E2L1 + H7N6F1E1L3 + H7N6F1E2L2) / (H3N4 + H3N3E1 + H3N4F1 + H4N4 + H3N5 + H3N3F1E1 + H4N3E1 + H4N4F1 + H5N4 + H3N5F1 + H4N5 + H4N3F1E1 + H4N4L1 + H5N4F1 + H4N4E1 + H4N5F1 + H5N5 + H5N4L1 + H4N4F1E1 + H5N4E1 + H5N5F1 + H4N5E1 + H5N4F1L1 + H4N7 + H5N4F1E1 + H4N5F1E1 + H5N5E1 + H5N4L2 + H5N4E1L1 + H5N4E2 + H5N5F1E1 + H6N5E1 + H5N4F1L2 + H4N6F1E1 + H5N4F1E1L1 + H4N7E1 + H5N4F1E2 + H6N5F1E1 + H5N5E2 + H6N5L2 + H5N5F1E1L1 + H6N5E1L1 + H5N5F1E2 + H6N5E2 + H6N5F1L2 + H6N5F1E1L1 + H6N5F1E2 + H6N5E1L2 + H6N5E2L1 + H6N5E3 + H6N5F1E1L2 + H6N5F1E2L1 + H6N5F1E3 + H6N5F2E2L1 + H7N6E1L2 + H7N6E2L1 + H7N6F1E1L2 + H7N6F1E2L1 + H7N6E1L3 + H7N6E2L2 + H7N6E3L1 + H7N6F1E1L3 + H7N6F1E2L2)
**CFa**	Relative abundance of species with 2 fucoses (i.e. at least one antennary fucose) within all complex type glycans	CFa = (H6N5F2E2L1) / (H3N4 + H3N3E1 + H3N4F1 + H4N4 + H3N5 + H3N3F1E1 + H4N3E1 + H4N4F1 + H5N4 + H3N5F1 + H4N5 + H4N3F1E1 + H4N4L1 + H5N4F1 + H4N4E1 + H4N5F1 + H5N5 + H5N4L1 + H4N4F1E1 + H5N4E1 + H5N5F1 + H4N5E1 + H5N4F1L1 + H4N7 + H5N4F1E1 + H4N5F1E1 + H5N5E1 + H5N4L2 + H5N4E1L1 + H5N4E2 + H5N5F1E1 + H6N5E1 + H5N4F1L2 + H4N6F1E1 + H5N4F1E1L1 + H4N7E1 + H5N4F1E2 + H6N5F1E1 + H5N5E2 + H6N5L2 + H5N5F1E1L1 + H6N5E1L1 + H5N5F1E2 + H6N5E2 + H6N5F1L2 + H6N5F1E1L1 + H6N5F1E2 + H6N5E1L2 + H6N5E2L1 + H6N5E3 + H6N5F1E1L2 + H6N5F1E2L1 + H6N5F1E3 + H6N5F2E2L1 + H7N6E1L2 + H7N6E2L1 + H7N6F1E1L2 + H7N6F1E2L1 + H7N6E1L3 + H7N6E2L2 + H7N6E3L1 + H7N6F1E1L3 + H7N6F1E2L2)

**A1F0**	Afucosylated monoantennary glycans	A1F0 = (H3N3E1 + H4N3E1) / (H3N3E1 + H3N3F1E1 + H4N3E1 + H4N3F1E1)
**A2F0**	Afucosylated diantennary glycans	A2F0 = (H3N4 + H4N4 + H3N5 + H5N4 + H4N5 + H4N4L1 + H4N4E1 + H5N5 + H5N4L1 + H5N4E1 + H4N5E1 + H5N5E1 + H5N4L2 + H5N4E1L1 + H5N4E2 + H5N5E2) / (H3N4 + H3N4F1 + H4N4 + H3N5 + H4N4F1 + H5N4 + H3N5F1 + H4N5 + H4N4L1 + H5N4F1 + H4N4E1 + H4N5F1 + H5N5 + H5N4L1 + H4N4F1E1 + H5N4E1 + H5N5F1 + H4N5E1 + H5N4F1L1 + H5N4F1E1 + H4N5F1E1 + H5N5E1 + H5N4L2 + H5N4E1L1 + H5N4E2 + H5N5F1E1 + H5N4F1L2 + H5N4F1E1L1 + H5N4F1E2 + H5N5E2 + H5N5F1E1L1 + H5N5F1E2)
**A3F0**	Afucosylated triantennary glycans	A3F0 = (H6N5E1 + H6N5L2 + H6N5E1L1 + H6N5E2 + H6N5E1L2 + H6N5E2L1 + H6N5E3) / (H6N5E1 + H6N5F1E1 + H6N5L2 + H6N5E1L1 + H6N5E2 + H6N5F1L2 + H6N5F1E1L1 + H6N5F1E2 + H6N5E1L2 + H6N5E2L1 + H6N5E3 + H6N5F1E1L2 + H6N5F1E2L1 + H6N5F1E3 + H6N5F2E2L1)
**A4F0**	Afucosylated tetra-antennary glycans	A4F0 = (H7N6E1L2 + H7N6E2L1 + H7N6E1L3 + H7N6E2L2 + H7N6E3L1) / (H4N6F1E1 + H7N6E1L2 + H7N6E2L1 + H7N6F1E1L2 + H7N6F1E2L1 + H7N6E1L3 + H7N6E2L2 + H7N6E3L1 + H7N6F1E1L3 + H7N6F1E2L2)
**A1F**	Fucosylation within monoantennary glycans	A1F = (H3N3F1E1 + H4N3F1E1) / (H3N3E1 + H3N3F1E1 + H4N3E1 + H4N3F1E1)
**A2F**	Fucosylation within diantennary glycans	A2F = (H3N4F1 + H4N4F1 + H3N5F1 + H5N4F1 + H4N5F1 + H4N4F1E1 + H5N5F1 + H5N4F1L1 + H5N4F1E1 + H4N5F1E1 + H5N5F1E1 + H5N4F1L2 + H5N4F1E1L1 + H5N4F1E2 + H5N5F1E1L1 + H5N5F1E2) / (H3N4 + H3N4F1 + H4N4 + H3N5 + H4N4F1 + H5N4 + H3N5F1 + H4N5 + H4N4L1 + H5N4F1 + H4N4E1 + H4N5F1 + H5N5 + H5N4L1 + H4N4F1E1 + H5N4E1 + H5N5F1 + H4N5E1 + H5N4F1L1 + H5N4F1E1 + H4N5F1E1 + H5N5E1 + H5N4L2 + H5N4E1L1 + H5N4E2 + H5N5F1E1 + H5N4F1L2 + H5N4F1E1L1 + H5N4F1E2 + H5N5E2 + H5N5F1E1L1 + H5N5F1E2)
**A3F**	Fucosylation within triantennary glycans	A3F = (H6N5F1E1 + H6N5F1L2 + H6N5F1E1L1 + H6N5F1E2 + H6N5F1E1L2 + H6N5F1E2L1 + H6N5F1E3 + H6N5F2E2L1) / (H6N5E1 + H6N5F1E1 + H6N5L2 + H6N5E1L1 + H6N5E2 + H6N5F1L2 + H6N5F1E1L1 + H6N5F1E2 + H6N5E1L2 + H6N5E2L1 + H6N5E3 + H6N5F1E1L2 + H6N5F1E2L1 + H6N5F1E3 + H6N5F2E2L1)
**A4F**	Fucosylation within tetra-antennary glycans	A4F = (H4N6F1E1 + H7N6F1E1L2 + H7N6F1E2L1 + H7N6F1E1L3 + H7N6F1E2L2) / (H4N6F1E1 + H7N6E1L2 + H7N6E2L1 + H7N6F1E1L2 + H7N6F1E2L1 + H7N6E1L3 + H7N6E2L2 + H7N6E3L1 + H7N6F1E1L3 + H7N6F1E2L2)
**A3Fa**	Relative abundance of species with 2 fucoses (i.e. at least one antennary fucose) within triantennary glycans	A3Fa = (H6N5F2E2L1) / (H6N5E1 + H6N5F1E1 + H6N5L2 + H6N5E1L1 + H6N5E2 + H6N5F1L2 + H6N5F1E1L1 + H6N5F1E2 + H6N5E1L2 + H6N5E2L1 + H6N5E3 + H6N5F1E1L2 + H6N5F1E2L1 + H6N5F1E3 + H6N5F2E2L1)

**A2S0F**	Fucosylation within non-sialylated diantennary glycans	A2S0F = (H3N4F1 + H4N4F1 + H3N5F1 + H5N4F1 + H4N5F1 + H5N5F1) / (H3N4 + H3N4F1 + H4N4 + H3N5 + H4N4F1 + H5N4 + H3N5F1 + H4N5 + H5N4F1 + H4N5F1 + H5N5 + H5N5F1)
**A1L0F**	Fucosylation within monoantennary glycans without α2,3-linked sialic acid	A1L0F = (H3N3F1E1 + H4N3F1E1) / (H3N3E1 + H3N3F1E1 + H4N3E1 + H4N3F1E1)
**A2L0F**	Fucosylation within diantennary glycans without α2,3-linked sialic acid	A2L0F = (H3N4F1 + H4N4F1 + H3N5F1 + H5N4F1 + H4N5F1 + H4N4F1E1 + H5N5F1 + H5N4F1E1 + H4N5F1E1 + H5N5F1E1 + H5N4F1E2 + H5N5F1E2) / (H3N4 + H3N4F1 + H4N4 + H3N5 + H4N4F1 + H5N4 + H3N5F1 + H4N5 + H5N4F1 + H4N4E1 + H4N5F1 + H5N5 + H4N4F1E1 + H5N4E1 + H5N5F1 + H4N5E1 + H5N4F1E1 + H4N5F1E1 + H5N5E1 + H5N4E2 + H5N5F1E1 + H5N4F1E2 + H5N5E2 + H5N5F1E2)
**A3L0F**	Fucosylation within triantennary glycans without α2,3-linked sialic acid	A3L0F = (H6N5F1E1 + H6N5F1E2 + H6N5F1E3) / (H6N5E1 + H6N5F1E1 + H6N5E2 + H6N5F1E2 + H6N5E3 + H6N5F1E3)
**A2E0F**	Fucosylation within diantennary glycans without α2,6-linked sialic acid	A2E0F = (H3N4F1 + H4N4F1 + H3N5F1 + H5N4F1 + H4N5F1 + H5N5F1 + H5N4F1L1 + H5N4F1L2) / (H3N4 + H3N4F1 + H4N4 + H3N5 + H4N4F1 + H5N4 + H3N5F1 + H4N5 + H4N4L1 + H5N4F1 + H4N5F1 + H5N5 + H5N4L1 + H5N5F1 + H5N4F1L1 + H5N4L2 + H5N4F1L2)
**A3E0F**	Fucosylation within triantennary glycans without α2,6-linked sialic acid	A3E0F = (H6N5F1L2) / (H6N5L2 + H6N5F1L2)
**A1SF**	Fucosylation within sialylated monoantennary glycans	A1SF = (H3N3F1E1 + H4N3F1E1) / (H3N3E1 + H3N3F1E1 + H4N3E1 + H4N3F1E1)
**A2SF**	Fucosylation within sialylated diantennary glycans	A2SF = (H4N4F1E1 + H5N4F1L1 + H5N4F1E1 + H4N5F1E1 + H5N5F1E1 + H5N4F1L2 + H5N4F1E1L1 + H5N4F1E2 + H5N5F1E1L1 + H5N5F1E2) / (H4N4L1 + H4N4E1 + H5N4L1 + H4N4F1E1 + H5N4E1 + H4N5E1 + H5N4F1L1 + H5N4F1E1 + H4N5F1E1 + H5N5E1 + H5N4L2 + H5N4E1L1 + H5N4E2 + H5N5F1E1 + H5N4F1L2 + H5N4F1E1L1 + H5N4F1E2 + H5N5E2 + H5N5F1E1L1 + H5N5F1E2)

**A3SF**	Fucosylation within sialylated triantennary glycans	A3SF = (H6N5F1E1 + H6N5F1L2 + H6N5F1E1L1 + H6N5F1E2 + H6N5F1E1L2 + H6N5F1E2L1 + H6N5F1E3 + H6N5F2E2L1) / (H6N5E1 + H6N5F1E1 + H6N5L2 + H6N5E1L1 + H6N5E2 + H6N5F1L2 + H6N5F1E1L1 + H6N5F1E2 + H6N5E1L2 + H6N5E2L1 + H6N5E3 + H6N5F1E1L2 + H6N5F1E2L1 + H6N5F1E3 + H6N5F2E2L1)
**A4SF**	Fucosylation within sialylated tetra-antennary glycans	A4SF = (H4N6F1E1 + H7N6F1E1L2 + H7N6F1E2L1 + H7N6F1E1L3 + H7N6F1E2L2) / (H4N6F1E1 + H7N6E1L2 + H7N6E2L1 + H7N6F1E1L2 + H7N6F1E2L1 + H7N6E1L3 + H7N6E2L2 + H7N6E3L1 + H7N6F1E1L3 + H7N6F1E2L2)
**A2LF**	Fucosylation within diantennary glycans with α2,3-linked sialic acid	A2LF = (H5N4F1L1 + H5N4F1L2 + H5N4F1E1L1 + H5N5F1E1L1) / (H4N4L1 + H5N4L1 + H5N4F1L1 + H5N4L2 + H5N4E1L1 + H5N4F1L2 + H5N4F1E1L1 + H5N5F1E1L1)
**A3LF**	Fucosylation within triantennary glycans with α2,3-linked sialic acid	A3LF = (H6N5F1L2 + H6N5F1E1L1 + H6N5F1E1L2 + H6N5F1E2L1 + H6N5F2E2L1) / (H6N5L2 + H6N5E1L1 + H6N5F1L2 + H6N5F1E1L1 + H6N5E1L2 + H6N5E2L1 + H6N5F1E1L2 + H6N5F1E2L1 + H6N5F2E2L1)
**A4LF**	Fucosylation within tetra-antennary glycans with α2,3-linked sialic acid	A4LF = (H7N6F1E1L2 + H7N6F1E2L1 + H7N6F1E1L3 + H7N6F1E2L2) / (H7N6E1L2 + H7N6E2L1 + H7N6F1E1L2 + H7N6F1E2L1 + H7N6E1L3 + H7N6E2L2 + H7N6E3L1 + H7N6F1E1L3 + H7N6F1E2L2)
**A1EF**	Fucosylation within monoantennary glycans with α2,6-linked sialic acid	A1EF = (H3N3F1E1 + H4N3F1E1) / (H3N3E1 + H3N3F1E1 + H4N3E1 + H4N3F1E1)
**A2EF**	Fucosylation within diantennary glycans with α2,6-linked sialic acid	A2EF = (H4N4F1E1 + H5N4F1E1 + H4N5F1E1 + H5N5F1E1 + H5N4F1E1L1 + H5N4F1E2 + H5N5F1E1L1 + H5N5F1E2) / (H4N4E1 + H4N4F1E1 + H5N4E1 + H4N5E1 + H5N4F1E1 + H4N5F1E1 + H5N5E1 + H5N4E1L1 + H5N4E2 + H5N5F1E1 + H5N4F1E1L1 + H5N4F1E2 + H5N5E2 + H5N5F1E1L1 + H5N5F1E2)
**A3EF**	Fucosylation within triantennary glycans with α2,6-linked sialic acid	A3EF = (H6N5F1E1 + H6N5F1E1L1 + H6N5F1E2 + H6N5F1E1L2 + H6N5F1E2L1 + H6N5F1E3 + H6N5F2E2L1) / (H6N5E1 + H6N5F1E1 + H6N5E1L1 + H6N5E2 + H6N5F1E1L1 + H6N5F1E2 + H6N5E1L2 + H6N5E2L1 + H6N5E3 + H6N5F1E1L2 + H6N5F1E2L1 + H6N5F1E3 + H6N5F2E2L1)
**A4EF**	Fucosylation within tetra-antennary glycans with α2,6-linked sialic acid	A4EF = (H4N6F1E1 + H7N6F1E1L2 + H7N6F1E2L1 + H7N6F1E1L3 + H7N6F1E2L2) / (H4N6F1E1 + H7N6E1L2 + H7N6E2L1 + H7N6F1E1L2 + H7N6F1E2L1 + H7N6E1L3 + H7N6E2L2 + H7N6E3L1 + H7N6F1E1L3 + H7N6F1E2L2)

**Bisection**		
**CB**	Relative abundance of species with a bisecting GlcNAc within all complex glycans	CB = (H3N5 + H3N5F1 + H4N5 + H4N5F1 + H5N5 + H5N5F1 + H4N5E1 + H4N5F1E1 + H5N5E1 + H5N5F1E1 + H5N5E2 + H5N5F1E1L1 + H5N5F1E2) / (H3N4 + H3N3E1 + H3N4F1 + H4N4 + H3N5 + H3N3F1E1 + H4N3E1 + H4N4F1 + H5N4 + H3N5F1 + H4N5 + H4N3F1E1 + H4N4L1 + H5N4F1 + H4N4E1 + H4N5F1 + H5N5 + H5N4L1 + H4N4F1E1 + H5N4E1 + H5N5F1 + H4N5E1 + H5N4F1L1 + H4N7 + H5N4F1E1 + H4N5F1E1 + H5N5E1 + H5N4L2 + H5N4E1L1 + H5N4E2 + H5N5F1E1 + H6N5E1 + H5N4F1L2 + H4N6F1E1 + H5N4F1E1L1 + H4N7E1 + H5N4F1E2 + H6N5F1E1 + H5N5E2 + H6N5L2 + H5N5F1E1L1 + H6N5E1L1 + H5N5F1E2 + H6N5E2 + H6N5F1L2 + H6N5F1E1L1 + H6N5F1E2 + H6N5E1L2 + H6N5E2L1 + H6N5E3 + H6N5F1E1L2 + H6N5F1E2L1 + H6N5F1E3 + H6N5F2E2L1 + H7N6E1L2 + H7N6E2L1 + H7N6F1E1L2 + H7N6F1E2L1 + H7N6E1L3 + H7N6E2L2 + H7N6E3L1 + H7N6F1E1L3 + H7N6F1E2L2)
**A2B**	Relative abundance of species with a bisecting GlcNAc within diantennary glycans	A2B = (H3N5 + H3N5F1 + H4N5 + H4N5F1 + H5N5 + H5N5F1 + H4N5E1 + H4N5F1E1 + H5N5E1 + H5N5F1E1 + H5N5E2 + H5N5F1E1L1 + H5N5F1E2) / (H3N4 + H3N4F1 + H4N4 + H3N5 + H4N4F1 + H5N4 + H3N5F1 + H4N5 + H4N4L1 + H5N4F1 + H4N4E1 + H4N5F1 + H5N5 + H5N4L1 + H4N4F1E1 + H5N4E1 + H5N5F1 + H4N5E1 + H5N4F1L1 + H5N4F1E1 + H4N5F1E1 + H5N5E1 + H5N4L2 + H5N4E1L1 + H5N4E2 + H5N5F1E1 + H5N4F1L2 + H5N4F1E1L1 + H5N4F1E2 + H5N5E2 + H5N5F1E1L1 + H5N5F1E2)
**A2F0B**	Relative abundance of species with a bisecting GlcNAc within non-fucosylated diantennary glycans	A2F0B = (H3N5 + H4N5 + H5N5 + H4N5E1 + H5N5E1 + H5N5E2) / (H3N4 + H4N4 + H3N5 + H5N4 + H4N5 + H4N4L1 + H4N4E1 + H5N5 + H5N4L1 + H5N4E1 + H4N5E1 + H5N5E1 + H5N4L2 + H5N4E1L1 + H5N4E2 + H5N5E2)
**A2FB**	Relative abundance of species with a bisecting GlcNAc within fucosylated diantennary	A2FB = (H3N5F1 + H4N5F1 + H5N5F1 + H4N5F1E1 + H5N5F1E1 + H5N5F1E1L1 + H5N5F1E2) / (H3N4F1 + H4N4F1 + H3N5F1 + H5N4F1 + H4N5F1 + H4N4F1E1 + H5N5F1 + H5N4F1L1 + H5N4F1E1 + H4N5F1E1 + H5N5F1E1 + H5N4F1L2 + H5N4F1E1L1 + H5N4F1E2 + H5N5F1E1L1 + H5N5F1E2)
**A2S0B**	Relative abundance of species with a bisecting GlcNAc within non-sialylated diantennary glycans	A2S0B = (H3N5 + H3N5F1 + H4N5 + H4N5F1 + H5N5 + H5N5F1) / (H3N4 + H3N4F1 + H4N4 + H3N5 + H4N4F1 + H5N4 + H3N5F1 + H4N5 + H5N4F1 + H4N5F1 + H5N5 + H5N5F1)
**A2SB**	Relative abundance of species with a bisecting GlcNAc within sialylated diantennary glycans	A2SB = (H4N5E1 + H4N5F1E1 + H5N5E1 + H5N5F1E1 + H5N5E2 + H5N5F1E1L1 + H5N5F1E2) / (H4N4L1 + H4N4E1 + H5N4L1 + H4N4F1E1 + H5N4E1 + H4N5E1 + H5N4F1L1 + H5N4F1E1 + H4N5F1E1 + H5N5E1 + H5N4L2 + H5N4E1L1 + H5N4E2 + H5N5F1E1 + H5N4F1L2 + H5N4F1E1L1 + H5N4F1E2 + H5N5E2 + H5N5F1E1L1 + H5N5F1E2)

**A2F0S0B**	Relative abundance of species with a bisecting GlcNAc within non-fucosylated non-sialylated diantennary glycans	A2F0S0B = (H3N5 + H4N5 + H5N5) / (H3N4 + H4N4 + H3N5 + H5N4 + H4N5 + H5N5)
**A2F0SB**	Relative abundance of species with a bisecting GlcNAc within non-fucosylated sialylated diantennary glycans	A2F0SB = (H4N5E1 + H5N5E1 + H5N5E2) / (H4N4L1 + H4N4E1 + H5N4L1 + H5N4E1 + H4N5E1 + H5N5E1 + H5N4L2 + H5N4E1L1 + H5N4E2 + H5N5E2)
**A2FS0B**	Relative abundance of species with a bisecting GlcNAc within fucosylated non-sialylated diantennary glycans	A2FS0B = (H3N5F1 + H4N5F1 + H5N5F1) / (H3N4F1 + H4N4F1 + H3N5F1 + H5N4F1 + H4N5F1 + H5N5F1)
**A2FSB**	Relative abundance of species with a bisecting GlcNAc within fucosylated sialylated diantennary glycans	A2FSB = (H4N5F1E1 + H5N5F1E1 + H5N5F1E1L1 + H5N5F1E2) / (H4N4F1E1 + H5N4F1L1 + H5N4F1E1 + H4N5F1E1 + H5N5F1E1 + H5N4F1L2 + H5N4F1E1L1 + H5N4F1E2 + H5N5F1E1L1 + H5N5F1E2)
**Galactosylation**		
**CG**	Galactosylation within all complex glycans	CG = (H3N3E1 + H4N4 + H3N3F1E1 + H4N3E1 + H4N4F1 + H5N4 + H4N5 + H4N3F1E1 + H4N4L1 + H5N4F1 + H4N4E1 + H4N5F1 + H5N5 + H5N4L1 + H4N4F1E1 + H5N4E1 + H5N5F1 + H4N5E1 + H5N4F1L1 + H5N4F1E1 + H4N5F1E1 + H5N5E1 + H5N4L2 + H5N4E1L1 + H5N4E2 + H5N5F1E1 + H6N5E1 + H5N4F1L2 + H4N6F1E1 + H5N4F1E1L1 + H4N7E1 + H5N4F1E2 + H6N5F1E1 + H5N5E2 + H6N5L2 + H5N5F1E1L1 + H6N5E1L1 + H5N5F1E2 + H6N5E2 + H6N5F1L2 + H6N5F1E1L1 + H6N5F1E2 + H6N5E1L2 + H6N5E2L1 + H6N5E3 + H6N5F1E1L2 + H6N5F1E2L1 + H6N5F1E3 + H6N5F2E2L1 + H7N6E1L2 + H7N6E2L1 + H7N6F1E1L2 + H7N6F1E2L1 + H7N6E1L3 + H7N6E2L2 + H7N6E3L1 + H7N6F1E1L3 + H7N6F1E2L2) / (H3N4 + H3N3E1 + H3N4F1 + H4N4 + H3N5 + H3N3F1E1 + H4N3E1 + H4N4F1 + H5N4 + H3N5F1 + H4N5 + H4N3F1E1 + H4N4L1 + H5N4F1 + H4N4E1 + H4N5F1 + H5N5 + H5N4L1 + H4N4F1E1 + H5N4E1 + H5N5F1 + H4N5E1 + H5N4F1L1 + H4N7 + H5N4F1E1 + H4N5F1E1 + H5N5E1 + H5N4L2 + H5N4E1L1 + H5N4E2 + H5N5F1E1 + H6N5E1 + H5N4F1L2 + H4N6F1E1 + H5N4F1E1L1 + H4N7E1 + H5N4F1E2 + H6N5F1E1 + H5N5E2 + H6N5L2 + H5N5F1E1L1 + H6N5E1L1 + H5N5F1E2 + H6N5E2 + H6N5F1L2 + H6N5F1E1L1 + H6N5F1E2 + H6N5E1L2 + H6N5E2L1 + H6N5E3 + H6N5F1E1L2 + H6N5F1E2L1 + H6N5F1E3 + H6N5F2E2L1 + H7N6E1L2 + H7N6E2L1 + H7N6F1E1L2 + H7N6F1E2L1 + H7N6E1L3 + H7N6E2L2 + H7N6E3L1 + H7N6F1E1L3 + H7N6F1E2L2)

**A2G**	Galactosylation per antenna within diantennary glycans	A2G = (0/2 * (H3N4 + H3N4F1 + H3N5 + H3N5F1) + 1/2 * (H4N4 + H4N4F1 + H4N5 + H4N4L1 + H4N4E1 + H4N5F1 + H4N4F1E1 + H4N5E1 + H4N5F1E1) + 2/2 * (H5N4 + H5N4F1 + H5N5 + H5N4L1 + H5N4E1 + H5N5F1 + H5N4F1L1 + H5N4F1E1 + H5N5E1 + H5N4L2 + H5N4E1L1 + H5N4E2 + H5N5F1E1 + H5N4F1L2 + H5N4F1E1L1 + H5N4F1E2 + H5N5E2 + H5N5F1E1L1 + H5N5F1E2)) / (H3N4 + H3N4F1 + H4N4 + H3N5 + H4N4F1 + H5N4 + H3N5F1 + H4N5 + H4N4L1 + H5N4F1 + H4N4E1 + H4N5F1 + H5N5 + H5N4L1 + H4N4F1E1 + H5N4E1 + H5N5F1 + H4N5E1 + H5N4F1L1 + H5N4F1E1 + H4N5F1E1 + H5N5E1 + H5N4L2 + H5N4E1L1 + H5N4E2 + H5N5F1E1 + H5N4F1L2 + H5N4F1E1L1 + H5N4F1E2 + H5N5E2 + H5N5F1E1L1 + H5N5F1E2)
**A4G**	Galactosylation per antenna within tetra-antennary glycans	A4G = (0/4 * (0) + 1/4 * (H4N6F1E1) + 2/4 * (0) + 3/4 * (0) + 4/4 * (H7N6E1L2 + H7N6E2L1 + H7N6F1E1L2 + H7N6F1E2L1 + H7N6E1L3 + H7N6E2L2 + H7N6E3L1 + H7N6F1E1L3 + H7N6F1E2L2)) / (H4N6F1E1 + H7N6E1L2 + H7N6E2L1 + H7N6F1E1L2 + H7N6F1E2L1 + H7N6E1L3 + H7N6E2L2 + H7N6E3L1 + H7N6F1E1L3 + H7N6F1E2L2)
**A2F0G**	Galactosylation per antenna within non-fucosylated diantennary glycans	A2F0G = (0/2 * (H3N4 + H3N5) + 1/2 * (H4N4 + H4N5 + H4N4L1 + H4N4E1 + H4N5E1) + 2/2 * (H5N4 + H5N5 + H5N4L1 + H5N4E1 + H5N5E1 + H5N4L2 + H5N4E1L1 + H5N4E2 + H5N5E2)) / (H3N4 + H4N4 + H3N5 + H5N4 + H4N5 + H4N4L1 + H4N4E1 + H5N5 + H5N4L1 + H5N4E1 + H4N5E1 + H5N5E1 + H5N4L2 + H5N4E1L1 + H5N4E2 + H5N5E2)
**A4F0G**	Galactosylation per antenna within non-fucosylated tetra-antennary glycans	A4F0G = (0/4 * (0) + 1/4 * (0) + 2/4 * (0) + 3/4 * (0) + 4/4 * (H7N6E1L2 + H7N6E2L1 + H7N6E1L3 + H7N6E2L2 + H7N6E3L1)) / (H4N6F1E1 + H7N6E1L2 + H7N6E2L1 + H7N6F1E1L2 + H7N6F1E2L1 + H7N6E1L3 + H7N6E2L2 + H7N6E3L1 + H7N6F1E1L3 + H7N6F1E2L2)
**A2FG**	Galactosylation per antenna within fucosylated diantennary glycans	A2FG = (0/2 * (H3N4F1 + H3N5F1) + 1/2 * (H4N4F1 + H4N5F1 + H4N4F1E1 + H4N5F1E1) + 2/2 * (H5N4F1 + H5N5F1 + H5N4F1L1 + H5N4F1E1 + H5N5F1E1 + H5N4F1L2 + H5N4F1E1L1 + H5N4F1E2 + H5N5F1E1L1 + H5N5F1E2)) / (H3N4F1 + H4N4F1 + H3N5F1 + H5N4F1 + H4N5F1 + H4N4F1E1 + H5N5F1 + H5N4F1L1 + H5N4F1E1 + H4N5F1E1 + H5N5F1E1 + H5N4F1L2 + H5N4F1E1L1 + H5N4F1E2 + H5N5F1E1L1 + H5N5F1E2)
**A4FG**	Galactosylation per antenna within fucosylated tetra-antennary glycans	A4FG = (0/4 * (0) + 1/4 * (H4N6F1E1) + 2/4 * (0) + 3/4 * (0) + 4/4 * (H7N6F1E1L2 + H7N6F1E2L1 + H7N6F1E1L3 + H7N6F1E2L2)) / (H4N6F1E1 + H7N6E1L2 + H7N6E2L1 + H7N6F1E1L2 + H7N6F1E2L1 + H7N6E1L3 + H7N6E2L2 + H7N6E3L1 + H7N6F1E1L3 + H7N6F1E2L2)
**A2S0G**	Galactosylation per antenna within non-sialylated diantennary glycans	A2S0G = (0/2 * (H3N4 + H3N4F1 + H3N5 + H3N5F1) + 1/2 * (H4N4 + H4N4F1 + H4N5 + H4N5F1) + 2/2 * (H5N4 + H5N4F1 + H5N5 + H5N5F1)) / (H3N4 + H3N4F1 + H4N4 + H3N5 + H4N4F1 + H5N4 + H3N5F1 + H4N5 + H5N4F1 + H4N5F1 + H5N5 + H5N5F1)
**A2SG**	Galactosylation per antenna within sialylated diantennary glycans	A2SG = (0/2 * (0) + 1/2 * (H4N4L1 + H4N4E1 + H4N4F1E1 + H4N5E1 + H4N5F1E1) + 2/2 * (H5N4L1 + H5N4E1 + H5N4F1L1 + H5N4F1E1 + H5N5E1 + H5N4L2 + H5N4E1L1 + H5N4E2 + H5N5F1E1 + H5N4F1L2 + H5N4F1E1L1 + H5N4F1E2 + H5N5E2 + H5N5F1E1L1 + H5N5F1E2)) / (H4N4L1 + H4N4E1 + H5N4L1 + H4N4F1E1 + H5N4E1 + H4N5E1 + H5N4F1L1 + H5N4F1E1 + H4N5F1E1 + H5N5E1 + H5N4L2 + H5N4E1L1 + H5N4E2 + H5N5F1E1 + H5N4F1L2 + H5N4F1E1L1 + H5N4F1E2 + H5N5E2 + H5N5F1E1L1 + H5N5F1E2)

**A2F0S0G**	Galactosylation per antenna within non-fucosylated, non-sialylated diantennary glycans	A2F0S0G = (0/2 * (H3N4 + H3N5) + 1/2 * (H4N4 + H4N5) + 2/2 * (H5N4 + H5N5)) / (H3N4 + H4N4 + H3N5 + H5N4 + H4N5 + H5N5)
**A2FS0G**	Galactosylation per antenna within fucosylated non-sialylated diantennary glycans	A2FS0G = (0/2 * (H3N4F1 + H3N5F1) + 1/2 * (H4N4F1 + H4N5F1) + 2/2 * (H5N4F1 + H5N5F1)) / (H3N4F1 + H4N4F1 + H3N5F1 + H5N4F1 + H4N5F1 + H5N5F1)
**A2F0SG**	Galactosylation per antenna within non-fucosylated sialylated diantennary glycans	A2F0SG = (0/2 * (0) + 1/2 * (H4N4L1 + H4N4E1 + H4N5E1) + 2/2 * (H5N4L1 + H5N4E1 + H5N5E1 + H5N4L2 + H5N4E1L1 + H5N4E2 + H5N5E2)) / (H4N4L1 + H4N4E1 + H5N4L1 + H5N4E1 + H4N5E1 + H5N5E1 + H5N4L2 + H5N4E1L1 + H5N4E2 + H5N5E2)
**A2FSG**	Galactosylation per antenna within fucosylated sialylated diantennary glycans	A2FSG = (0/2 * (0) + 1/2 * (H4N4F1E1 + H4N5F1E1) + 2/2 * (H5N4F1L1 + H5N4F1E1 + H5N5F1E1 + H5N4F1L2 + H5N4F1E1L1 + H5N4F1E2 + H5N5F1E1L1 + H5N5F1E2)) / (H4N4F1E1 + H5N4F1L1 + H5N4F1E1 + H4N5F1E1 + H5N5F1E1 + H5N4F1L2 + H5N4F1E1L1 + H5N4F1E2 + H5N5F1E1L1 + H5N5F1E2)
**Sialylation**		
**CS**	Sialylation per antenna within all complex glycans	CS = (H3N3E1 + H3N3F1E1 + H4N3E1 + H4N3F1E1 + H4N4L1 + H4N4E1 + H5N4L1 + H4N4F1E1 + H5N4E1 + H4N5E1 + H5N4F1L1 + H5N4F1E1 + H4N5F1E1 + H5N5E1 + H5N4L2 + H5N4E1L1 + H5N4E2 + H5N5F1E1 + H6N5E1 + H5N4F1L2 + H4N6F1E1 + H5N4F1E1L1 + H4N7E1 + H5N4F1E2 + H6N5F1E1 + H5N5E2 + H6N5L2 + H5N5F1E1L1 + H6N5E1L1 + H5N5F1E2 + H6N5E2 + H6N5F1L2 + H6N5F1E1L1 + H6N5F1E2 + H6N5E1L2 + H6N5E2L1 + H6N5E3 + H6N5F1E1L2 + H6N5F1E2L1 + H6N5F1E3 + H6N5F2E2L1 + H7N6E1L2 + H7N6E2L1 + H7N6F1E1L2 + H7N6F1E2L1 + H7N6E1L3 + H7N6E2L2 + H7N6E3L1 + H7N6F1E1L3 + H7N6F1E2L2) / (H3N4 + H3N3E1 + H3N4F1 + H4N4 + H3N5 + H3N3F1E1 + H4N3E1 + H4N4F1 + H5N4 + H3N5F1 + H4N5 + H4N3F1E1 + H4N4L1 + H5N4F1 + H4N4E1 + H4N5F1 + H5N5 + H5N4L1 + H4N4F1E1 + H5N4E1 + H5N5F1 + H4N5E1 + H5N4F1L1 + H4N7 + H5N4F1E1 + H4N5F1E1 + H5N5E1 + H5N4L2 + H5N4E1L1 + H5N4E2 + H5N5F1E1 + H6N5E1 + H5N4F1L2 + H4N6F1E1 + H5N4F1E1L1 + H4N7E1 + H5N4F1E2 + H6N5F1E1 + H5N5E2 + H6N5L2 + H5N5F1E1L1 + H6N5E1L1 + H5N5F1E2 + H6N5E2 + H6N5F1L2 + H6N5F1E1L1 + H6N5F1E2 + H6N5E1L2 + H6N5E2L1 + H6N5E3 + H6N5F1E1L2 + H6N5F1E2L1 + H6N5F1E3 + H6N5F2E2L1 + H7N6E1L2 + H7N6E2L1 + H7N6F1E1L2 + H7N6F1E2L1 + H7N6E1L3 + H7N6E2L2 + H7N6E3L1 + H7N6F1E1L3 + H7N6F1E2L2)

**A2S**	Sialylation per antenna within diantennary glycans	A2S = (0/2 * (H3N4 + H3N4F1 + H4N4 + H3N5 + H4N4F1 + H5N4 + H3N5F1 + H4N5 + H5N4F1 + H4N5F1 + H5N5 + H5N5F1) + 1/2 * (H4N4L1 + H4N4E1 + H5N4L1 + H4N4F1E1 + H5N4E1 + H4N5E1 + H5N4F1L1 + H5N4F1E1 + H4N5F1E1 + H5N5E1 + H5N5F1E1) + 2/2 * (H5N4L2 + H5N4E1L1 + H5N4E2 + H5N4F1L2 + H5N4F1E1L1 + H5N4F1E2 + H5N5E2 + H5N5F1E1L1 + H5N5F1E2)) / (H3N4 + H3N4F1 + H4N4 + H3N5 + H4N4F1 + H5N4 + H3N5F1 + H4N5 + H4N4L1 + H5N4F1 + H4N4E1 + H4N5F1 + H5N5 + H5N4L1 + H4N4F1E1 + H5N4E1 + H5N5F1 + H4N5E1 + H5N4F1L1 + H5N4F1E1 + H4N5F1E1 + H5N5E1 + H5N4L2 + H5N4E1L1 + H5N4E2 + H5N5F1E1 + H5N4F1L2 + H5N4F1E1L1 + H5N4F1E2 + H5N5E2 + H5N5F1E1L1 + H5N5F1E2)
**A3S**	Sialylation per antenna within triantennary glycans	A3S = (0/3 * (0) + 1/3 * (H6N5E1 + H6N5F1E1) + 2/3 * (H6N5L2 + H6N5E1L1 + H6N5E2 + H6N5F1L2 + H6N5F1E1L1 + H6N5F1E2) + 3/3 * (H6N5E1L2 + H6N5E2L1 + H6N5E3 + H6N5F1E1L2 + H6N5F1E2L1 + H6N5F1E3 + H6N5F2E2L1)) / (H6N5E1 + H6N5F1E1 + H6N5L2 + H6N5E1L1 + H6N5E2 + H6N5F1L2 + H6N5F1E1L1 + H6N5F1E2 + H6N5E1L2 + H6N5E2L1 + H6N5E3 + H6N5F1E1L2 + H6N5F1E2L1 + H6N5F1E3 + H6N5F2E2L1)
**A4S**	Sialylation per antenna within tetra-antennary glycans	A4S = (0/4 * (0) + 1/4 * (H4N6F1E1) + 2/4 * (0) + 3/4 * (H7N6E1L2 + H7N6E2L1 + H7N6F1E1L2 + H7N6F1E2L1) + 4/4 * (H7N6E1L3 + H7N6E2L2 + H7N6E3L1 + H7N6F1E1L3 + H7N6F1E2L2)) / (H4N6F1E1 + H7N6E1L2 + H7N6E2L1 + H7N6F1E1L2 + H7N6F1E2L1 + H7N6E1L3 + H7N6E2L2 + H7N6E3L1 + H7N6F1E1L3 + H7N6F1E2L2)
**A2F0S**	Sialylation per antenna within non-fucosylated diantennary glycans	A2F0S = (0/2 * (H3N4 + H4N4 + H3N5 + H5N4 + H4N5 + H5N5) + 1/2 * (H4N4L1 + H4N4E1 + H5N4L1 + H5N4E1 + H4N5E1 + H5N5E1) + 2/2 * (H5N4L2 + H5N4E1L1 + H5N4E2 + H5N5E2)) / (H3N4 + H4N4 + H3N5 + H5N4 + H4N5 + H4N4L1 + H4N4E1 + H5N5 + H5N4L1 + H5N4E1 + H4N5E1 + H5N5E1 + H5N4L2 + H5N4E1L1 + H5N4E2 + H5N5E2)
**A3F0S**	Sialylation per antenna within non-fucosylated triantennary glycans	A3F0S = (0/3 * (0) + 1/3 * (H6N5E1) + 2/3 * (H6N5L2 + H6N5E1L1 + H6N5E2) + 3/3 * (H6N5E1L2 + H6N5E2L1 + H6N5E3)) / (H6N5E1 + H6N5L2 + H6N5E1L1 + H6N5E2 + H6N5E1L2 + H6N5E2L1 + H6N5E3)
**A4F0S**	Sialylation per antenna within non-fucosylated tetra-antennary glycans	A4F0S = (0/4 * (0) + 1/4 * (0) + 2/4 * (0) + 3/4 * (H7N6E1L2 + H7N6E2L1) + 4/4 * (H7N6E1L3 + H7N6E2L2 + H7N6E3L1)) / (H4N6F1E1 + H7N6E1L2 + H7N6E2L1 + H7N6F1E1L2 + H7N6F1E2L1 + H7N6E1L3 + H7N6E2L2 + H7N6E3L1 + H7N6F1E1L3 + H7N6F1E2L2)
**A2FS**	Sialylation per antenna within fucosylated diantennary glycans	A2FS = (0/2 * (H3N4F1 + H4N4F1 + H3N5F1 + H5N4F1 + H4N5F1 + H5N5F1) + 1/2 * (H4N4F1E1 + H5N4F1L1 + H5N4F1E1 + H4N5F1E1 + H5N5F1E1) + 2/2 * (H5N4F1L2 + H5N4F1E1L1 + H5N4F1E2 + H5N5F1E1L1 + H5N5F1E2)) / (H3N4F1 + H4N4F1 + H3N5F1 + H5N4F1 + H4N5F1 + H4N4F1E1 + H5N5F1 + H5N4F1L1 + H5N4F1E1 + H4N5F1E1 + H5N5F1E1 + H5N4F1L2 + H5N4F1E1L1 + H5N4F1E2 + H5N5F1E1L1 + H5N5F1E2)
**A3FS**	Sialylation per antenna within fucosylated triantennary glycans	A3FS = (0/3 * (0) + 1/3 * (H6N5F1E1) + 2/3 * (H6N5F1L2 + H6N5F1E1L1 + H6N5F1E2) + 3/3 * (H6N5F1E1L2 + H6N5F1E2L1 + H6N5F1E3 + H6N5F2E2L1)) / (H6N5F1E1 + H6N5F1L2 + H6N5F1E1L1 + H6N5F1E2 + H6N5F1E1L2 + H6N5F1E2L1 + H6N5F1E3 + H6N5F2E2L1)

**A4FS**	Sialylation per antenna within fucosylated tetra-antennary glycans	A4FS = (0/4 * (0) + 1/4 * (H4N6F1E1) + 2/4 * (0) + 3/4 * (H7N6F1E1L2 + H7N6F1E2L1) + 4/4 * (H7N6F1E1L3 + H7N6F1E2L2)) / (H4N6F1E1 + H7N6E1L2 + H7N6E2L1 + H7N6F1E1L2 + H7N6F1E2L1 + H7N6E1L3 + H7N6E2L2 + H7N6E3L1 + H7N6F1E1L3 + H7N6F1E2L2)
**A2GS**	Sialylation per galactose within diantennary glycans	A2GS = ((0/2 * (H3N4 + H3N4F1 + H4N4 + H3N5 + H4N4F1 + H5N4 + H3N5F1 + H4N5 + H5N4F1 + H4N5F1 + H5N5 + H5N5F1) + 1/2 * (H4N4L1 + H4N4E1 + H5N4L1 + H4N4F1E1 + H5N4E1 + H4N5E1 + H5N4F1L1 + H5N4F1E1 + H4N5F1E1 + H5N5E1 + H5N5F1E1) + 2/2 * (H5N4L2 + H5N4E1L1 + H5N4E2 + H5N4F1L2 + H5N4F1E1L1 + H5N4F1E2 + H5N5E2 + H5N5F1E1L1 + H5N5F1E2)) / (H3N4 + H3N4F1 + H4N4 + H3N5 + H4N4F1 + H5N4 + H3N5F1 + H4N5 + H4N4L1 + H5N4F1 + H4N4E1 + H4N5F1 + H5N5 + H5N4L1 + H4N4F1E1 + H5N4E1 + H5N5F1 + H4N5E1 + H5N4F1L1 + H5N4F1E1 + H4N5F1E1 + H5N5E1 + H5N4L2 + H5N4E1L1 + H5N4E2 + H5N5F1E1 + H5N4F1L2 + H5N4F1E1L1 + H5N4F1E2 + H5N5E2 + H5N5F1E1L1 + H5N5F1E2)) / ((0/2 * (H3N4 + H3N4F1 + H3N5 + H3N5F1) + 1/2 * (H4N4 + H4N4F1 + H4N5 + H4N4L1 + H4N4E1 + H4N5F1 + H4N4F1E1 + H4N5E1 + H4N5F1E1) + 2/2 * (H5N4 + H5N4F1 + H5N5 + H5N4L1 + H5N4E1 + H5N5F1 + H5N4F1L1 + H5N4F1E1 + H5N5E1 + H5N4L2 + H5N4E1L1 + H5N4E2 + H5N5F1E1 + H5N4F1L2 + H5N4F1E1L1 + H5N4F1E2 + H5N5E2 + H5N5F1E1L1 + H5N5F1E2)) / (H3N4 + H3N4F1 + H4N4 + H3N5 + H4N4F1 + H5N4 + H3N5F1 + H4N5 + H4N4L1 + H5N4F1 + H4N4E1 + H4N5F1 + H5N5 + H5N4L1 + H4N4F1E1 + H5N4E1 + H5N5F1 + H4N5E1 + H5N4F1L1 + H5N4F1E1 + H4N5F1E1 + H5N5E1 + H5N4L2 + H5N4E1L1 + H5N4E2 + H5N5F1E1 + H5N4F1L2 + H5N4F1E1L1 + H5N4F1E2 + H5N5E2 + H5N5F1E1L1 + H5N5F1E2))
**A3GS**	Sialylation per galactose within triantennary glycans	A3GS = ((0/3 * (0) + 1/3 * (H6N5E1 + H6N5F1E1) + 2/3 * (H6N5L2 + H6N5E1L1 + H6N5E2 + H6N5F1L2 + H6N5F1E1L1 + H6N5F1E2) + 3/3 * (H6N5E1L2 + H6N5E2L1 + H6N5E3 + H6N5F1E1L2 + H6N5F1E2L1 + H6N5F1E3 + H6N5F2E2L1)) / (H6N5E1 + H6N5F1E1 + H6N5L2 + H6N5E1L1 + H6N5E2 + H6N5F1L2 + H6N5F1E1L1 + H6N5F1E2 + H6N5E1L2 + H6N5E2L1 + H6N5E3 + H6N5F1E1L2 + H6N5F1E2L1 + H6N5F1E3 + H6N5F2E2L1)) / ((0/3 * (0) + 1/3 * (0) + 2/3 * (0) + 3/3 * (H6N5E1 + H6N5F1E1 + H6N5L2 + H6N5E1L1 + H6N5E2 + H6N5F1L2 + H6N5F1E1L1 + H6N5F1E2 + H6N5E1L2 + H6N5E2L1 + H6N5E3 + H6N5F1E1L2 + H6N5F1E2L1 + H6N5F1E3 + H6N5F2E2L1)) / (H6N5E1 + H6N5F1E1 + H6N5L2 + H6N5E1L1 + H6N5E2 + H6N5F1L2 + H6N5F1E1L1 + H6N5F1E2 + H6N5E1L2 + H6N5E2L1 + H6N5E3 + H6N5F1E1L2 + H6N5F1E2L1 + H6N5F1E3 + H6N5F2E2L1))
**A4GS**	Sialylation per galactose within tetra-antennary glycans	A4GS = ((0/4 * (0) + 1/4 * (H4N6F1E1) + 2/4 * (0) + 3/4 * (H7N6E1L2 + H7N6E2L1 + H7N6F1E1L2 + H7N6F1E2L1) + 4/4 * (H7N6E1L3 + H7N6E2L2 + H7N6E3L1 + H7N6F1E1L3 + H7N6F1E2L2)) / (H4N6F1E1 + H7N6E1L2 + H7N6E2L1 + H7N6F1E1L2 + H7N6F1E2L1 + H7N6E1L3 + H7N6E2L2 + H7N6E3L1 + H7N6F1E1L3 + H7N6F1E2L2)) / ((0/4 * (0) + 1/4 * (H4N6F1E1) + 2/4 * (0) + 3/4 * (0) + 4/4 * (H7N6E1L2 + H7N6E2L1 + H7N6F1E1L2 + H7N6F1E2L1 + H7N6E1L3 + H7N6E2L2 + H7N6E3L1 + H7N6F1E1L3 + H7N6F1E2L2)) / (H4N6F1E1 + H7N6E1L2 + H7N6E2L1 + H7N6F1E1L2 + H7N6F1E2L1 + H7N6E1L3 + H7N6E2L2 + H7N6E3L1 + H7N6F1E1L3 + H7N6F1E2L2))
**A2F0GS**	Sialylation per galactose within non-fucosylated diantennary glycans	A2F0GS = ((0/2 * (H3N4 + H4N4 + H3N5 + H5N4 + H4N5 + H5N5) + 1/2 * (H4N4L1 + H4N4E1 + H5N4L1 + H5N4E1 + H4N5E1 + H5N5E1) + 2/2 * (H5N4L2 + H5N4E1L1 + H5N4E2 + H5N5E2)) / (H3N4 + H4N4 + H3N5 + H5N4 + H4N5 + H4N4L1 + H4N4E1 + H5N5 + H5N4L1 + H5N4E1 + H4N5E1 + H5N5E1 + H5N4L2 + H5N4E1L1 + H5N4E2 + H5N5E2)) / ((0/2 * (H3N4 + H3N5) + 1/2 * (H4N4 + H4N5 + H4N4L1 + H4N4E1 + H4N5E1) + 2/2 * (H5N4 + H5N5 + H5N4L1 + H5N4E1 + H5N5E1 + H5N4L2 + H5N4E1L1 + H5N4E2 + H5N5E2)) / (H3N4 + H4N4 + H3N5 + H5N4 + H4N5 + H4N4L1 + H4N4E1 + H5N5 + H5N4L1 + H5N4E1 + H4N5E1 + H5N5E1 + H5N4L2 + H5N4E1L1 + H5N4E2 + H5N5E2))

**A3F0GS**	Sialylation per galactose within non-fucosylated triantennary glycans	A3F0GS = ((0/3 * (0) + 1/3 * (H6N5E1) + 2/3 * (H6N5L2 + H6N5E1L1 + H6N5E2) + 3/3 * (H6N5E1L2 + H6N5E2L1 + H6N5E3)) / (H6N5E1 + H6N5L2 + H6N5E1L1 + H6N5E2 + H6N5E1L2 + H6N5E2L1 + H6N5E3)) / ((0/3 * (0) + 1/3 * (0) + 2/3 * (0) + 3/3 * (H6N5E1 + H6N5L2 + H6N5E1L1 + H6N5E2 + H6N5E1L2 + H6N5E2L1 + H6N5E3)) / (H6N5E1 + H6N5L2 + H6N5E1L1 + H6N5E2 + H6N5E1L2 + H6N5E2L1 + H6N5E3))
**A4F0GS**	Sialylation per galactose within non-fucosylated tetra-antennary glycans	A4F0GS = ((0/4 * (0) + 1/4 * (0) + 2/4 * (0) + 3/4 * (H7N6E1L2 + H7N6E2L1) + 4/4 * (H7N6E1L3 + H7N6E2L2 + H7N6E3L1)) / (H4N6F1E1 + H7N6E1L2 + H7N6E2L1 + H7N6F1E1L2 + H7N6F1E2L1 + H7N6E1L3 + H7N6E2L2 + H7N6E3L1 + H7N6F1E1L3 + H7N6F1E2L2)) / ((0/4 * (0) + 1/4 * (0) + 2/4 * (0) + 3/4 * (0) + 4/4 * (H7N6E1L2 + H7N6E2L1 + H7N6E1L3 + H7N6E2L2 + H7N6E3L1)) / (H4N6F1E1 + H7N6E1L2 + H7N6E2L1 + H7N6F1E1L2 + H7N6F1E2L1 + H7N6E1L3 + H7N6E2L2 + H7N6E3L1 + H7N6F1E1L3 + H7N6F1E2L2))
**A2FGS**	Sialylation per galactose within fucosylated diantennary glycans	A2FGS = ((0/2 * (H3N4F1 + H4N4F1 + H3N5F1 + H5N4F1 + H4N5F1 + H5N5F1) + 1/2 * (H4N4F1E1 + H5N4F1L1 + H5N4F1E1 + H4N5F1E1 + H5N5F1E1) + 2/2 * (H5N4F1L2 + H5N4F1E1L1 + H5N4F1E2 + H5N5F1E1L1 + H5N5F1E2)) / (H3N4F1 + H4N4F1 + H3N5F1 + H5N4F1 + H4N5F1 + H4N4F1E1 + H5N5F1 + H5N4F1L1 + H5N4F1E1 + H4N5F1E1 + H5N5F1E1 + H5N4F1L2 + H5N4F1E1L1 + H5N4F1E2 + H5N5F1E1L1 + H5N5F1E2)) / ((0/2 * (H3N4F1 + H3N5F1) + 1/2 * (H4N4F1 + H4N5F1 + H4N4F1E1 + H4N5F1E1) + 2/2 * (H5N4F1 + H5N5F1 + H5N4F1L1 + H5N4F1E1 + H5N5F1E1 + H5N4F1L2 + H5N4F1E1L1 + H5N4F1E2 + H5N5F1E1L1 + H5N5F1E2)) / (H3N4F1 + H4N4F1 + H3N5F1 + H5N4F1 + H4N5F1 + H4N4F1E1 + H5N5F1 + H5N4F1L1 + H5N4F1E1 + H4N5F1E1 + H5N5F1E1 + H5N4F1L2 + H5N4F1E1L1 + H5N4F1E2 + H5N5F1E1L1 + H5N5F1E2))
**A3FGS**	Sialylation per galactose within fucosylated triantennary glycans	A3FGS = ((0/3 * (0) + 1/3 * (H6N5F1E1) + 2/3 * (H6N5F1L2 + H6N5F1E1L1 + H6N5F1E2) + 3/3 * (H6N5F1E1L2 + H6N5F1E2L1 + H6N5F1E3 + H6N5F2E2L1)) / (H6N5F1E1 + H6N5F1L2 + H6N5F1E1L1 + H6N5F1E2 + H6N5F1E1L2 + H6N5F1E2L1 + H6N5F1E3 + H6N5F2E2L1)) / ((0/3 * (0) + 1/3 * (0) + 2/3 * (0) + 3/3 * (H6N5F1E1 + H6N5F1L2 + H6N5F1E1L1 + H6N5F1E2 + H6N5F1E1L2 + H6N5F1E2L1 + H6N5F1E3 + H6N5F2E2L1)) / (H6N5F1E1 + H6N5F1L2 + H6N5F1E1L1 + H6N5F1E2 + H6N5F1E1L2 + H6N5F1E2L1 + H6N5F1E3 + H6N5F2E2L1))
**A4FGS**	Sialylation per galactose within fucosylated tetra-antennary glycans	A4FGS = ((0/4 * (0) + 1/4 * (H4N6F1E1) + 2/4 * (0) + 3/4 * (H7N6F1E1L2 + H7N6F1E2L1) + 4/4 * (H7N6F1E1L3 + H7N6F1E2L2)) / (H4N6F1E1 + H7N6E1L2 + H7N6E2L1 + H7N6F1E1L2 + H7N6F1E2L1 + H7N6E1L3 + H7N6E2L2 + H7N6E3L1 + H7N6F1E1L3 + H7N6F1E2L2)) / ((0/4 * (0) + 1/4 * (H4N6F1E1) + 2/4 * (0) + 3/4 * (0) + 4/4 * (H7N6F1E1L2 + H7N6F1E2L1 + H7N6F1E1L3 + H7N6F1E2L2)) / (H4N6F1E1 + H7N6E1L2 + H7N6E2L1 + H7N6F1E1L2 + H7N6F1E2L1 + H7N6E1L3 + H7N6E2L2 + H7N6E3L1 + H7N6F1E1L3 + H7N6F1E2L2))
**α2,3-linked sialylation (L)**		
**A2L**	α2,3-sialylation per antenna within diantennary glycans	A2L = (0/2 * (H3N4 + H3N4F1 + H4N4 + H3N5 + H4N4F1 + H5N4 + H3N5F1 + H4N5 + H5N4F1 + H4N4E1 + H4N5F1 + H5N5 + H4N4F1E1 + H5N4E1 + H5N5F1 + H4N5E1 + H5N4F1E1 + H4N5F1E1 + H5N5E1 + H5N4E2 + H5N5F1E1 + H5N4F1E2 + H5N5E2 + H5N5F1E2) + 1/2 * (H4N4L1 + H5N4L1 + H5N4F1L1 + H5N4E1L1 + H5N4F1E1L1 + H5N5F1E1L1) + 2/2 * (H5N4L2 + H5N4F1L2)) / (H3N4 + H3N4F1 + H4N4 + H3N5 + H4N4F1 + H5N4 + H3N5F1 + H4N5 + H4N4L1 + H5N4F1 + H4N4E1 + H4N5F1 + H5N5 + H5N4L1 + H4N4F1E1 + H5N4E1 + H5N5F1 + H4N5E1 + H5N4F1L1 + H5N4F1E1 + H4N5F1E1 + H5N5E1 + H5N4L2 + H5N4E1L1 + H5N4E2 + H5N5F1E1 + H5N4F1L2 + H5N4F1E1L1 + H5N4F1E2 + H5N5E2 + H5N5F1E1L1 + H5N5F1E2)

**A3L**	α2,3-sialylation per antenna within triantennary glycans	A3L = (0/3 * (H6N5E1 + H6N5F1E1 + H6N5E2 + H6N5F1E2 + H6N5E3 + H6N5F1E3) + 1/3 * (H6N5E1L1 + H6N5F1E1L1 + H6N5E2L1 + H6N5F1E2L1 + H6N5F2E2L1) + 2/3 * (H6N5L2 + H6N5F1L2 + H6N5E1L2 + H6N5F1E1L2) + 3/3 * (0)) / (H6N5E1 + H6N5F1E1 + H6N5L2 + H6N5E1L1 + H6N5E2 + H6N5F1L2 + H6N5F1E1L1 + H6N5F1E2 + H6N5E1L2 + H6N5E2L1 + H6N5E3 + H6N5F1E1L2 + H6N5F1E2L1 + H6N5F1E3 + H6N5F2E2L1)
**A4L**	α2,3-sialylation per antenna within tetra-antennary glycans	A4L = (0/4 * (H4N6F1E1) + 1/4 * (H7N6E2L1 + H7N6F1E2L1 + H7N6E3L1) + 2/4 * (H7N6E1L2 + H7N6F1E1L2 + H7N6E2L2 + H7N6F1E2L2) + 3/4 * (H7N6E1L3 + H7N6F1E1L3) + 4/4 * (0)) / (H4N6F1E1 + H7N6E1L2 + H7N6E2L1 + H7N6F1E1L2 + H7N6F1E2L1 + H7N6E1L3 + H7N6E2L2 + H7N6E3L1 + H7N6F1E1L3 + H7N6F1E2L2)
**A2F0L**	α2,3-sialylation per antenna within non-fucosylated diantennary glycans	A2F0L = (0/2 * (H3N4 + H4N4 + H3N5 + H5N4 + H4N5 + H4N4E1 + H5N5 + H5N4E1 + H4N5E1 + H5N5E1 + H5N4E2 + H5N5E2) + 1/2 * (H4N4L1 + H5N4L1 + H5N4E1L1) + 2/2 * (H5N4L2)) / (H3N4 + H4N4 + H3N5 + H5N4 + H4N5 + H4N4L1 + H4N4E1 + H5N5 + H5N4L1 + H5N4E1 + H4N5E1 + H5N5E1 + H5N4L2 + H5N4E1L1 + H5N4E2 + H5N5E2)
**A3F0L**	α2,3-sialylation per antenna within non-fucosylated triantennary glycans	A3F0L = (0/3 * (H6N5E1 + H6N5E2 + H6N5E3) + 1/3 * (H6N5E1L1 + H6N5E2L1) + 2/3 * (H6N5L2 + H6N5E1L2) + 3/3 * (0)) / (H6N5E1 + H6N5L2 + H6N5E1L1 + H6N5E2 + H6N5E1L2 + H6N5E2L1 + H6N5E3)
**A4F0L**	α2,3-sialylation per antenna within non-fucosylated tetra-antennary glycans	A4F0L = (0/4 * (0) + 1/4 * (H7N6E2L1 + H7N6E3L1) + 2/4 * (H7N6E1L2 + H7N6E2L2) + 3/4 * (H7N6E1L3) + 4/4 * (0)) / (H4N6F1E1 + H7N6E1L2 + H7N6E2L1 + H7N6F1E1L2 + H7N6F1E2L1 + H7N6E1L3 + H7N6E2L2 + H7N6E3L1 + H7N6F1E1L3 + H7N6F1E2L2)
**A2FL**	α2,3-sialylation per antenna within fucosylated diantennary glycans	A2FL = (0/2 * (H3N4F1 + H4N4F1 + H3N5F1 + H5N4F1 + H4N5F1 + H4N4F1E1 + H5N5F1 + H5N4F1E1 + H4N5F1E1 + H5N5F1E1 + H5N4F1E2 + H5N5F1E2) + 1/2 * (H5N4F1L1 + H5N4F1E1L1 + H5N5F1E1L1) + 2/2 * (H5N4F1L2)) / (H3N4F1 + H4N4F1 + H3N5F1 + H5N4F1 + H4N5F1 + H4N4F1E1 + H5N5F1 + H5N4F1L1 + H5N4F1E1 + H4N5F1E1 + H5N5F1E1 + H5N4F1L2 + H5N4F1E1L1 + H5N4F1E2 + H5N5F1E1L1 + H5N5F1E2)
**A3FL**	α2,3-sialylation per antenna within fucosylated triantennary glycans	A3FL = (0/3 * (H6N5F1E1 + H6N5F1E2 + H6N5F1E3) + 1/3 * (H6N5F1E1L1 + H6N5F1E2L1 + H6N5F2E2L1) + 2/3 * (H6N5F1L2 + H6N5F1E1L2) + 3/3 * (0)) / (H6N5F1E1 + H6N5F1L2 + H6N5F1E1L1 + H6N5F1E2 + H6N5F1E1L2 + H6N5F1E2L1 + H6N5F1E3 + H6N5F2E2L1)
**A4FL**	α2,3-sialylation per antenna within fucosylated tetra-antennary glycans	A4FL = (0/4 * (H4N6F1E1) + 1/4 * (H7N6F1E2L1) + 2/4 * (H7N6F1E1L2 + H7N6F1E2L2) + 3/4 * (H7N6F1E1L3) + 4/4 * (0)) / (H4N6F1E1 + H7N6E1L2 + H7N6E2L1 + H7N6F1E1L2 + H7N6F1E2L1 + H7N6E1L3 + H7N6E2L2 + H7N6E3L1 + H7N6F1E1L3 + H7N6F1E2L2)

**A2GL**	α2,3-sialylation per galactose within diantennary glycans	A2GL = ((0/2 * (H3N4 + H3N4F1 + H4N4 + H3N5 + H4N4F1 + H5N4 + H3N5F1 + H4N5 + H5N4F1 + H4N4E1 + H4N5F1 + H5N5 + H4N4F1E1 + H5N4E1 + H5N5F1 + H4N5E1 + H5N4F1E1 + H4N5F1E1 + H5N5E1 + H5N4E2 + H5N5F1E1 + H5N4F1E2 + H5N5E2 + H5N5F1E2) + 1/2 * (H4N4L1 + H5N4L1 + H5N4F1L1 + H5N4E1L1 + H5N4F1E1L1 + H5N5F1E1L1) + 2/2 * (H5N4L2 + H5N4F1L2)) / (H3N4 + H3N4F1 + H4N4 + H3N5 + H4N4F1 + H5N4 + H3N5F1 + H4N5 + H4N4L1 + H5N4F1 + H4N4E1 + H4N5F1 + H5N5 + H5N4L1 + H4N4F1E1 + H5N4E1 + H5N5F1 + H4N5E1 + H5N4F1L1 + H5N4F1E1 + H4N5F1E1 + H5N5E1 + H5N4L2 + H5N4E1L1 + H5N4E2 + H5N5F1E1 + H5N4F1L2 + H5N4F1E1L1 + H5N4F1E2 + H5N5E2 + H5N5F1E1L1 + H5N5F1E2)) / ((0/2 * (H3N4 + H3N4F1 + H3N5 + H3N5F1) + 1/2 * (H4N4 + H4N4F1 + H4N5 + H4N4L1 + H4N4E1 + H4N5F1 + H4N4F1E1 + H4N5E1 + H4N5F1E1) + 2/2 * (H5N4 + H5N4F1 + H5N5 + H5N4L1 + H5N4E1 + H5N5F1 + H5N4F1L1 + H5N4F1E1 + H5N5E1 + H5N4L2 + H5N4E1L1 + H5N4E2 + H5N5F1E1 + H5N4F1L2 + H5N4F1E1L1 + H5N4F1E2 + H5N5E2 + H5N5F1E1L1 + H5N5F1E2)) / (H3N4 + H3N4F1 + H4N4 + H3N5 + H4N4F1 + H5N4 + H3N5F1 + H4N5 + H4N4L1 + H5N4F1 + H4N4E1 + H4N5F1 + H5N5 + H5N4L1 + H4N4F1E1 + H5N4E1 + H5N5F1 + H4N5E1 + H5N4F1L1 + H5N4F1E1 + H4N5F1E1 + H5N5E1 + H5N4L2 + H5N4E1L1 + H5N4E2 + H5N5F1E1 + H5N4F1L2 + H5N4F1E1L1 + H5N4F1E2 + H5N5E2 + H5N5F1E1L1 + H5N5F1E2))
**A3GL**	α2,3-sialylation per galactose within triantennary glycans	A3GL = ((0/3 * (H6N5E1 + H6N5F1E1 + H6N5E2 + H6N5F1E2 + H6N5E3 + H6N5F1E3) + 1/3 * (H6N5E1L1 + H6N5F1E1L1 + H6N5E2L1 + H6N5F1E2L1 + H6N5F2E2L1) + 2/3 * (H6N5L2 + H6N5F1L2 + H6N5E1L2 + H6N5F1E1L2) + 3/3 * (0)) / (H6N5E1 + H6N5F1E1 + H6N5L2 + H6N5E1L1 + H6N5E2 + H6N5F1L2 + H6N5F1E1L1 + H6N5F1E2 + H6N5E1L2 + H6N5E2L1 + H6N5E3 + H6N5F1E1L2 + H6N5F1E2L1 + H6N5F1E3 + H6N5F2E2L1)) / ((0/3 * (0) + 1/3 * (0) + 2/3 * (0) + 3/3 * (H6N5E1 + H6N5F1E1 + H6N5L2 + H6N5E1L1 + H6N5E2 + H6N5F1L2 + H6N5F1E1L1 + H6N5F1E2 + H6N5E1L2 + H6N5E2L1 + H6N5E3 + H6N5F1E1L2 + H6N5F1E2L1 + H6N5F1E3 + H6N5F2E2L1)) / (H6N5E1 + H6N5F1E1 + H6N5L2 + H6N5E1L1 + H6N5E2 + H6N5F1L2 + H6N5F1E1L1 + H6N5F1E2 + H6N5E1L2 + H6N5E2L1 + H6N5E3 + H6N5F1E1L2 + H6N5F1E2L1 + H6N5F1E3 + H6N5F2E2L1))
**A4GL**	α2,3-sialylation per galactose within tetra-antennary glycans	A4GL = ((0/4 * (H4N6F1E1) + 1/4 * (H7N6E2L1 + H7N6F1E2L1 + H7N6E3L1) + 2/4 * (H7N6E1L2 + H7N6F1E1L2 + H7N6E2L2 + H7N6F1E2L2) + 3/4 * (H7N6E1L3 + H7N6F1E1L3) + 4/4 * (0)) / (H4N6F1E1 + H7N6E1L2 + H7N6E2L1 + H7N6F1E1L2 + H7N6F1E2L1 + H7N6E1L3 + H7N6E2L2 + H7N6E3L1 + H7N6F1E1L3 + H7N6F1E2L2)) / ((0/4 * (0) + 1/4 * (H4N6F1E1) + 2/4 * (0) + 3/4 * (0) + 4/4 * (H7N6E1L2 + H7N6E2L1 + H7N6F1E1L2 + H7N6F1E2L1 + H7N6E1L3 + H7N6E2L2 + H7N6E3L1 + H7N6F1E1L3 + H7N6F1E2L2)) / (H4N6F1E1 + H7N6E1L2 + H7N6E2L1 + H7N6F1E1L2 + H7N6F1E2L1 + H7N6E1L3 + H7N6E2L2 + H7N6E3L1 + H7N6F1E1L3 + H7N6F1E2L2))
**A2F0GL**	α2,3-sialylation per galactose within non-fucosylated diantennary glycans	A2F0GL = ((0/2 * (H3N4 + H4N4 + H3N5 + H5N4 + H4N5 + H4N4E1 + H5N5 + H5N4E1 + H4N5E1 + H5N5E1 + H5N4E2 + H5N5E2) + 1/2 * (H4N4L1 + H5N4L1 + H5N4E1L1) + 2/2 * (H5N4L2)) / (H3N4 + H4N4 + H3N5 + H5N4 + H4N5 + H4N4L1 + H4N4E1 + H5N5 + H5N4L1 + H5N4E1 + H4N5E1 + H5N5E1 + H5N4L2 + H5N4E1L1 + H5N4E2 + H5N5E2)) / ((0/2 * (H3N4 + H3N5) + 1/2 * (H4N4 + H4N5 + H4N4L1 + H4N4E1 + H4N5E1) + 2/2 * (H5N4 + H5N5 + H5N4L1 + H5N4E1 + H5N5E1 + H5N4L2 + H5N4E1L1 + H5N4E2 + H5N5E2)) / (H3N4 + H4N4 + H3N5 + H5N4 + H4N5 + H4N4L1 + H4N4E1 + H5N5 + H5N4L1 + H5N4E1 + H4N5E1 + H5N5E1 + H5N4L2 + H5N4E1L1 + H5N4E2 + H5N5E2))
**A3F0GL**	α2,3-sialylation per galactose within non-fucosylated triantennary glycans	A3F0GL = ((0/3 * (H6N5E1 + H6N5E2 + H6N5E3) + 1/3 * (H6N5E1L1 + H6N5E2L1) + 2/3 * (H6N5L2 + H6N5E1L2) + 3/3 * (0)) / (H6N5E1 + H6N5L2 + H6N5E1L1 + H6N5E2 + H6N5E1L2 + H6N5E2L1 + H6N5E3)) / ((0/3 * (0) + 1/3 * (0) + 2/3 * (0) + 3/3 * (H6N5E1 + H6N5L2 + H6N5E1L1 + H6N5E2 + H6N5E1L2 + H6N5E2L1 + H6N5E3)) / (H6N5E1 + H6N5L2 + H6N5E1L1 + H6N5E2 + H6N5E1L2 + H6N5E2L1 + H6N5E3))

**A4F0GL**	α2,3-sialylation per galactose within non-fucosylated tetra-antennary glycans	A4F0GL = ((0/4 * (0) + 1/4 * (H7N6E2L1 + H7N6E3L1) + 2/4 * (H7N6E1L2 + H7N6E2L2) + 3/4 * (H7N6E1L3) + 4/4 * (0)) / (H4N6F1E1 + H7N6E1L2 + H7N6E2L1 + H7N6F1E1L2 + H7N6F1E2L1 + H7N6E1L3 + H7N6E2L2 + H7N6E3L1 + H7N6F1E1L3 + H7N6F1E2L2)) / ((0/4 * (0) + 1/4 * (0) + 2/4 * (0) + 3/4 * (0) + 4/4 * (H7N6E1L2 + H7N6E2L1 + H7N6E1L3 + H7N6E2L2 + H7N6E3L1)) / (H4N6F1E1 + H7N6E1L2 + H7N6E2L1 + H7N6F1E1L2 + H7N6F1E2L1 + H7N6E1L3 + H7N6E2L2 + H7N6E3L1 + H7N6F1E1L3 + H7N6F1E2L2))
**A2FGL**	α2,3-sialylation per galactose within fucosylated diantennary glycans	A2FGL = ((0/2 * (H3N4F1 + H4N4F1 + H3N5F1 + H5N4F1 + H4N5F1 + H4N4F1E1 + H5N5F1 + H5N4F1E1 + H4N5F1E1 + H5N5F1E1 + H5N4F1E2 + H5N5F1E2) + 1/2 * (H5N4F1L1 + H5N4F1E1L1 + H5N5F1E1L1) + 2/2 * (H5N4F1L2)) / (H3N4F1 + H4N4F1 + H3N5F1 + H5N4F1 + H4N5F1 + H4N4F1E1 + H5N5F1 + H5N4F1L1 + H5N4F1E1 + H4N5F1E1 + H5N5F1E1 + H5N4F1L2 + H5N4F1E1L1 + H5N4F1E2 + H5N5F1E1L1 + H5N5F1E2)) / ((0/2 * (H3N4F1 + H3N5F1) + 1/2 * (H4N4F1 + H4N5F1 + H4N4F1E1 + H4N5F1E1) + 2/2 * (H5N4F1 + H5N5F1 + H5N4F1L1 + H5N4F1E1 + H5N5F1E1 + H5N4F1L2 + H5N4F1E1L1 + H5N4F1E2 + H5N5F1E1L1 + H5N5F1E2)) / (H3N4F1 + H4N4F1 + H3N5F1 + H5N4F1 + H4N5F1 + H4N4F1E1 + H5N5F1 + H5N4F1L1 + H5N4F1E1 + H4N5F1E1 + H5N5F1E1 + H5N4F1L2 + H5N4F1E1L1 + H5N4F1E2 + H5N5F1E1L1 + H5N5F1E2))
**A3FGL**	α2,3-sialylation per galactose within fucosylated triantennary glycans	A3FGL = ((0/3 * (H6N5F1E1 + H6N5F1E2 + H6N5F1E3) + 1/3 * (H6N5F1E1L1 + H6N5F1E2L1 + H6N5F2E2L1) + 2/3 * (H6N5F1L2 + H6N5F1E1L2) + 3/3 * (0)) / (H6N5F1E1 + H6N5F1L2 + H6N5F1E1L1 + H6N5F1E2 + H6N5F1E1L2 + H6N5F1E2L1 + H6N5F1E3 + H6N5F2E2L1)) / ((0/3 * (0) + 1/3 * (0) + 2/3 * (0) + 3/3 * (H6N5F1E1 + H6N5F1L2 + H6N5F1E1L1 + H6N5F1E2 + H6N5F1E1L2 + H6N5F1E2L1 + H6N5F1E3 + H6N5F2E2L1)) / (H6N5F1E1 + H6N5F1L2 + H6N5F1E1L1 + H6N5F1E2 + H6N5F1E1L2 + H6N5F1E2L1 + H6N5F1E3 + H6N5F2E2L1))
**A4FGL**	α2,3-sialylation per galactose within fucosylated tetra-antennary glycans	A4FGL = ((0/4 * (H4N6F1E1) + 1/4 * (H7N6F1E2L1) + 2/4 * (H7N6F1E1L2 + H7N6F1E2L2) + 3/4 * (H7N6F1E1L3) + 4/4 * (0)) / (H4N6F1E1 + H7N6E1L2 + H7N6E2L1 + H7N6F1E1L2 + H7N6F1E2L1 + H7N6E1L3 + H7N6E2L2 + H7N6E3L1 + H7N6F1E1L3 + H7N6F1E2L2)) / ((0/4 * (0) + 1/4 * (H4N6F1E1) + 2/4 * (0) + 3/4 * (0) + 4/4 * (H7N6F1E1L2 + H7N6F1E2L1 + H7N6F1E1L3 + H7N6F1E2L2)) / (H4N6F1E1 + H7N6E1L2 + H7N6E2L1 + H7N6F1E1L2 + H7N6F1E2L1 + H7N6E1L3 + H7N6E2L2 + H7N6E3L1 + H7N6F1E1L3 + H7N6F1E2L2))
**α2,6-linked sialylation (E)**		
**A2E**	α2,6-sialylation per antenna within diantennary glycans	A2E = (0/2 * (H3N4 + H3N4F1 + H4N4 + H3N5 + H4N4F1 + H5N4 + H3N5F1 + H4N5 + H4N4L1 + H5N4F1 + H4N5F1 + H5N5 + H5N4L1 + H5N5F1 + H5N4F1L1 + H5N4L2 + H5N4F1L2) + 1/2 * (H4N4E1 + H4N4F1E1 + H5N4E1 + H4N5E1 + H5N4F1E1 + H4N5F1E1 + H5N5E1 + H5N4E1L1 + H5N5F1E1 + H5N4F1E1L1 + H5N5F1E1L1) + 2/2 * (H5N4E2 + H5N4F1E2 + H5N5E2 + H5N5F1E2)) / (H3N4 + H3N4F1 + H4N4 + H3N5 + H4N4F1 + H5N4 + H3N5F1 + H4N5 + H4N4L1 + H5N4F1 + H4N4E1 + H4N5F1 + H5N5 + H5N4L1 + H4N4F1E1 + H5N4E1 + H5N5F1 + H4N5E1 + H5N4F1L1 + H5N4F1E1 + H4N5F1E1 + H5N5E1 + H5N4L2 + H5N4E1L1 + H5N4E2 + H5N5F1E1 + H5N4F1L2 + H5N4F1E1L1 + H5N4F1E2 + H5N5E2 + H5N5F1E1L1 + H5N5F1E2)
**A3E**	α2,6-sialylation per antenna within triantennary glycans	A3E = (0/3 * (H6N5L2 + H6N5F1L2) + 1/3 * (H6N5E1 + H6N5F1E1 + H6N5E1L1 + H6N5F1E1L1 + H6N5E1L2 + H6N5F1E1L2) + 2/3 * (H6N5E2 + H6N5F1E2 + H6N5E2L1 + H6N5F1E2L1 + H6N5F2E2L1) + 3/3 * (H6N5E3 + H6N5F1E3)) / (H6N5E1 + H6N5F1E1 + H6N5L2 + H6N5E1L1 + H6N5E2 + H6N5F1L2 + H6N5F1E1L1 + H6N5F1E2 + H6N5E1L2 + H6N5E2L1 + H6N5E3 + H6N5F1E1L2 + H6N5F1E2L1 + H6N5F1E3 + H6N5F2E2L1)

**A4E**	α2,6-sialylation per antenna within tetra-antennary glycans	A4E = (0/4 * (0) + 1/4 * (H4N6F1E1 + H7N6E1L2 + H7N6F1E1L2 + H7N6E1L3 + H7N6F1E1L3) + 2/4 * (H7N6E2L1 + H7N6F1E2L1 + H7N6E2L2 + H7N6F1E2L2) + 3/4 * (H7N6E3L1) + 4/4 * (0)) / (H4N6F1E1 + H7N6E1L2 + H7N6E2L1 + H7N6F1E1L2 + H7N6F1E2L1 + H7N6E1L3 + H7N6E2L2 + H7N6E3L1 + H7N6F1E1L3 + H7N6F1E2L2)
**A2F0E**	α2,6-sialylation per antenna within non-fucosylated diantennary glycans	A2F0E = (0/2 * (H3N4 + H4N4 + H3N5 + H5N4 + H4N5 + H4N4L1 + H5N5 + H5N4L1 + H5N4L2) + 1/2 * (H4N4E1 + H5N4E1 + H4N5E1 + H5N5E1 + H5N4E1L1) + 2/2 * (H5N4E2 + H5N5E2)) / (H3N4 + H4N4 + H3N5 + H5N4 + H4N5 + H4N4L1 + H4N4E1 + H5N5 + H5N4L1 + H5N4E1 + H4N5E1 + H5N5E1 + H5N4L2 + H5N4E1L1 + H5N4E2 + H5N5E2)
**A3F0E**	α2,6-sialylation per antenna within non-fucosylated triantennary glycans	A3F0E = (0/3 * (H6N5L2) + 1/3 * (H6N5E1 + H6N5E1L1 + H6N5E1L2) + 2/3 * (H6N5E2 + H6N5E2L1) + 3/3 * (H6N5E3)) / (H6N5E1 + H6N5L2 + H6N5E1L1 + H6N5E2 + H6N5E1L2 + H6N5E2L1 + H6N5E3)
**A4F0E**	α2,6-sialylation per antenna within non-fucosylated tetra-antennary glycans	A4F0E = (0/4 * (0) + 1/4 * (H7N6E1L2 + H7N6E1L3) + 2/4 * (H7N6E2L1 + H7N6E2L2) + 3/4 * (H7N6E3L1) + 4/4 * (0)) / (H4N6F1E1 + H7N6E1L2 + H7N6E2L1 + H7N6F1E1L2 + H7N6F1E2L1 + H7N6E1L3 + H7N6E2L2 + H7N6E3L1 + H7N6F1E1L3 + H7N6F1E2L2)
**A2FE**	α2,6-sialylation per antenna within fucosylated diantennary glycans	A2FE = (0/2 * (H3N4F1 + H4N4F1 + H3N5F1 + H5N4F1 + H4N5F1 + H5N5F1 + H5N4F1L1 + H5N4F1L2) + 1/2 * (H4N4F1E1 + H5N4F1E1 + H4N5F1E1 + H5N5F1E1 + H5N4F1E1L1 + H5N5F1E1L1) + 2/2 * (H5N4F1E2 + H5N5F1E2)) / (H3N4F1 + H4N4F1 + H3N5F1 + H5N4F1 + H4N5F1 + H4N4F1E1 + H5N5F1 + H5N4F1L1 + H5N4F1E1 + H4N5F1E1 + H5N5F1E1 + H5N4F1L2 + H5N4F1E1L1 + H5N4F1E2 + H5N5F1E1L1 + H5N5F1E2)
**A3FE**	α2,6-sialylation per antenna within fucosylated triantennary glycans	A3FE = (0/3 * (H6N5F1L2) + 1/3 * (H6N5F1E1 + H6N5F1E1L1 + H6N5F1E1L2) + 2/3 * (H6N5F1E2 + H6N5F1E2L1 + H6N5F2E2L1) + 3/3 * (H6N5F1E3)) / (H6N5F1E1 + H6N5F1L2 + H6N5F1E1L1 + H6N5F1E2 + H6N5F1E1L2 + H6N5F1E2L1 + H6N5F1E3 + H6N5F2E2L1)
**A4FE**	α2,6-sialylation per antenna within fucosylated tetra-antennary glycans	A4FE = (0/4 * (0) + 1/4 * (H4N6F1E1 + H7N6F1E1L2 + H7N6F1E1L3) + 2/4 * (H7N6F1E2L1 + H7N6F1E2L2) + 3/4 * (0) + 4/4 * (0)) / (H4N6F1E1 + H7N6E1L2 + H7N6E2L1 + H7N6F1E1L2 + H7N6F1E2L1 + H7N6E1L3 + H7N6E2L2 + H7N6E3L1 + H7N6F1E1L3 + H7N6F1E2L2)
**A2GE**	α2,6-sialylation per galactose within diantennary glycans	A2GE = ((0/2 * (H3N4 + H3N4F1 + H4N4 + H3N5 + H4N4F1 + H5N4 + H3N5F1 + H4N5 + H4N4L1 + H5N4F1 + H4N5F1 + H5N5 + H5N4L1 + H5N5F1 + H5N4F1L1 + H5N4L2 + H5N4F1L2) + 1/2 * (H4N4E1 + H4N4F1E1 + H5N4E1 + H4N5E1 + H5N4F1E1 + H4N5F1E1 + H5N5E1 + H5N4E1L1 + H5N5F1E1 + H5N4F1E1L1 + H5N5F1E1L1) + 2/2 * (H5N4E2 + H5N4F1E2 + H5N5E2 + H5N5F1E2)) / (H3N4 + H3N4F1 + H4N4 + H3N5 + H4N4F1 + H5N4 + H3N5F1 + H4N5 + H4N4L1 + H5N4F1 + H4N4E1 + H4N5F1 + H5N5 + H5N4L1 + H4N4F1E1 + H5N4E1 + H5N5F1 + H4N5E1 + H5N4F1L1 + H5N4F1E1 + H4N5F1E1 + H5N5E1 + H5N4L2 + H5N4E1L1 + H5N4E2 + H5N5F1E1 + H5N4F1L2 + H5N4F1E1L1 + H5N4F1E2 + H5N5E2 + H5N5F1E1L1 + H5N5F1E2)) / ((0/2 * (H3N4 + H3N4F1 + H3N5 + H3N5F1) + 1/2 * (H4N4 + H4N4F1 + H4N5 + H4N4L1 + H4N4E1 + H4N5F1 + H4N4F1E1 + H4N5E1 + H4N5F1E1) + 2/2 * (H5N4 + H5N4F1 + H5N5 + H5N4L1 + H5N4E1 + H5N5F1 + H5N4F1L1 + H5N4F1E1 + H5N5E1 + H5N4L2 + H5N4E1L1 + H5N4E2 + H5N5F1E1 + H5N4F1L2 + H5N4F1E1L1 + H5N4F1E2 + H5N5E2 + H5N5F1E1L1 + H5N5F1E2)) / (H3N4 + H3N4F1 + H4N4 + H3N5 + H4N4F1 + H5N4 + H3N5F1 +

		H4N5 + H4N4L1 + H5N4F1 + H4N4E1 + H4N5F1 + H5N5 + H5N4L1 + H4N4F1E1 + H5N4E1 + H5N5F1 + H4N5E1 + H5N4F1L1 + H5N4F1E1 + H4N5F1E1 + H5N5E1 + H5N4L2 + H5N4E1L1 + H5N4E2 + H5N5F1E1 + H5N4F1L2 + H5N4F1E1L1 + H5N4F1E2 + H5N5E2 + H5N5F1E1L1 + H5N5F1E2))
**A3GE**	α2,6-sialylation per galactose within triantennary glycans	A3GE = ((0/3 * (H6N5L2 + H6N5F1L2) + 1/3 * (H6N5E1 + H6N5F1E1 + H6N5E1L1 + H6N5F1E1L1 + H6N5E1L2 + H6N5F1E1L2) + 2/3 * (H6N5E2 + H6N5F1E2 + H6N5E2L1 + H6N5F1E2L1 + H6N5F2E2L1) + 3/3 * (H6N5E3 + H6N5F1E3)) / (H6N5E1 + H6N5F1E1 + H6N5L2 + H6N5E1L1 + H6N5E2 + H6N5F1L2 + H6N5F1E1L1 + H6N5F1E2 + H6N5E1L2 + H6N5E2L1 + H6N5E3 + H6N5F1E1L2 + H6N5F1E2L1 + H6N5F1E3 + H6N5F2E2L1)) / ((0/3 * (0) + 1/3 * (0) + 2/3 * (0) + 3/3 * (H6N5E1 + H6N5F1E1 + H6N5L2 + H6N5E1L1 + H6N5E2 + H6N5F1L2 + H6N5F1E1L1 + H6N5F1E2 + H6N5E1L2 + H6N5E2L1 + H6N5E3 + H6N5F1E1L2 + H6N5F1E2L1 + H6N5F1E3 + H6N5F2E2L1)) / (H6N5E1 + H6N5F1E1 + H6N5L2 + H6N5E1L1 + H6N5E2 + H6N5F1L2 + H6N5F1E1L1 + H6N5F1E2 + H6N5E1L2 + H6N5E2L1 + H6N5E3 + H6N5F1E1L2 + H6N5F1E2L1 + H6N5F1E3 + H6N5F2E2L1))
**A4GE**	α2,6-sialylation per galactose within tetra-antennary glycans	A4GE = ((0/4 * (0) + 1/4 * (H4N6F1E1 + H7N6E1L2 + H7N6F1E1L2 + H7N6E1L3 + H7N6F1E1L3) + 2/4 * (H7N6E2L1 + H7N6F1E2L1 + H7N6E2L2 + H7N6F1E2L2) + 3/4 * (H7N6E3L1) + 4/4 * (0)) / (H4N6F1E1 + H7N6E1L2 + H7N6E2L1 + H7N6F1E1L2 + H7N6F1E2L1 + H7N6E1L3 + H7N6E2L2 + H7N6E3L1 + H7N6F1E1L3 + H7N6F1E2L2)) / ((0/4 * (0) + 1/4 * (H4N6F1E1) + 2/4 * (0) + 3/4 * (0) + 4/4 * (H7N6E1L2 + H7N6E2L1 + H7N6F1E1L2 + H7N6F1E2L1 + H7N6E1L3 + H7N6E2L2 + H7N6E3L1 + H7N6F1E1L3 + H7N6F1E2L2)) / (H4N6F1E1 + H7N6E1L2 + H7N6E2L1 + H7N6F1E1L2 + H7N6F1E2L1 + H7N6E1L3 + H7N6E2L2 + H7N6E3L1 + H7N6F1E1L3 + H7N6F1E2L2))
**A2F0GE**	α2,6-sialylation per galactose within non-fucosylated diantennary glycans	A2F0GE = ((0/2 * (H3N4 + H4N4 + H3N5 + H5N4 + H4N5 + H4N4L1 + H5N5 + H5N4L1 + H5N4L2) + 1/2 * (H4N4E1 + H5N4E1 + H4N5E1 + H5N5E1 + H5N4E1L1) + 2/2 * (H5N4E2 + H5N5E2)) / (H3N4 + H4N4 + H3N5 + H5N4 + H4N5 + H4N4L1 + H4N4E1 + H5N5 + H5N4L1 + H5N4E1 + H4N5E1 + H5N5E1 + H5N4L2 + H5N4E1L1 + H5N4E2 + H5N5E2)) / ((0/2 * (H3N4 + H3N5) + 1/2 * (H4N4 + H4N5 + H4N4L1 + H4N4E1 + H4N5E1) + 2/2 * (H5N4 + H5N5 + H5N4L1 + H5N4E1 + H5N5E1 + H5N4L2 + H5N4E1L1 + H5N4E2 + H5N5E2)) / (H3N4 + H4N4 + H3N5 + H5N4 + H4N5 + H4N4L1 + H4N4E1 + H5N5 + H5N4L1 + H5N4E1 + H4N5E1 + H5N5E1 + H5N4L2 + H5N4E1L1 + H5N4E2 + H5N5E2))
**A3F0GE**	α2,6-sialylation per galactose within non-fucosylated triantennary glycans	A3F0GE = ((0/3 * (H6N5L2) + 1/3 * (H6N5E1 + H6N5E1L1 + H6N5E1L2) + 2/3 * (H6N5E2 + H6N5E2L1) + 3/3 * (H6N5E3)) / (H6N5E1 + H6N5L2 + H6N5E1L1 + H6N5E2 + H6N5E1L2 + H6N5E2L1 + H6N5E3)) / ((0/3 * (0) + 1/3 * (0) + 2/3 * (0) + 3/3 * (H6N5E1 + H6N5L2 + H6N5E1L1 + H6N5E2 + H6N5E1L2 + H6N5E2L1 + H6N5E3)) / (H6N5E1 + H6N5L2 + H6N5E1L1 + H6N5E2 + H6N5E1L2 + H6N5E2L1 + H6N5E3))
**A4F0GE**	α2,6-sialylation per galactose within non-fucosylated tetra-antennary glycans	A4F0GE = ((0/4 * (0) + 1/4 * (H7N6E1L2 + H7N6E1L3) + 2/4 * (H7N6E2L1 + H7N6E2L2) + 3/4 * (H7N6E3L1) + 4/4 * (0)) / (H4N6F1E1 + H7N6E1L2 + H7N6E2L1 + H7N6F1E1L2 + H7N6F1E2L1 + H7N6E1L3 + H7N6E2L2 + H7N6E3L1 + H7N6F1E1L3 + H7N6F1E2L2)) / ((0/4 * (0) + 1/4 * (0) + 2/4 * (0) + 3/4 * (0) + 4/4 * (H7N6E1L2 + H7N6E2L1 + H7N6E1L3 + H7N6E2L2 + H7N6E3L1)) / (H4N6F1E1 + H7N6E1L2 + H7N6E2L1 + H7N6F1E1L2 + H7N6F1E2L1 + H7N6E1L3 + H7N6E2L2 + H7N6E3L1 + H7N6F1E1L3 + H7N6F1E2L2))

**A2FGE**	α2,6-sialylation per galactose within fucosylated diantennary glycans	A2FGE = ((0/2 * (H3N4F1 + H4N4F1 + H3N5F1 + H5N4F1 + H4N5F1 + H5N5F1 + H5N4F1L1 + H5N4F1L2) + 1/2 * (H4N4F1E1 + H5N4F1E1 + H4N5F1E1 + H5N5F1E1 + H5N4F1E1L1 + H5N5F1E1L1) + 2/2 * (H5N4F1E2 + H5N5F1E2)) / (H3N4F1 + H4N4F1 + H3N5F1 + H5N4F1 + H4N5F1 + H4N4F1E1 + H5N5F1 + H5N4F1L1 + H5N4F1E1 + H4N5F1E1 + H5N5F1E1 + H5N4F1L2 + H5N4F1E1L1 + H5N4F1E2 + H5N5F1E1L1 + H5N5F1E2)) / ((0/2 * (H3N4F1 + H3N5F1) + 1/2 * (H4N4F1 + H4N5F1 + H4N4F1E1 + H4N5F1E1) + 2/2 * (H5N4F1 + H5N5F1 + H5N4F1L1 + H5N4F1E1 + H5N5F1E1 + H5N4F1L2 + H5N4F1E1L1 + H5N4F1E2 + H5N5F1E1L1 + H5N5F1E2)) / (H3N4F1 + H4N4F1 + H3N5F1 + H5N4F1 + H4N5F1 + H4N4F1E1 + H5N5F1 + H5N4F1L1 + H5N4F1E1 + H4N5F1E1 + H5N5F1E1 + H5N4F1L2 + H5N4F1E1L1 + H5N4F1E2 + H5N5F1E1L1 + H5N5F1E2))
**A3FGE**	α2,6-sialylation per galactose within fucosylated triantennary glycans	A3FGE = ((0/3 * (H6N5F1L2) + 1/3 * (H6N5F1E1 + H6N5F1E1L1 + H6N5F1E1L2) + 2/3 * (H6N5F1E2 + H6N5F1E2L1 + H6N5F2E2L1) + 3/3 * (H6N5F1E3)) / (H6N5F1E1 + H6N5F1L2 + H6N5F1E1L1 + H6N5F1E2 + H6N5F1E1L2 + H6N5F1E2L1 + H6N5F1E3 + H6N5F2E2L1)) / ((0/3 * (0) + 1/3 * (0) + 2/3 * (0) + 3/3 * (H6N5F1E1 + H6N5F1L2 + H6N5F1E1L1 + H6N5F1E2 + H6N5F1E1L2 + H6N5F1E2L1 + H6N5F1E3 + H6N5F2E2L1)) / (H6N5F1E1 + H6N5F1L2 + H6N5F1E1L1 + H6N5F1E2 + H6N5F1E1L2 + H6N5F1E2L1 + H6N5F1E3 + H6N5F2E2L1))
**A4FGE**	α2,6-sialylation per galactose within fucosylated tetra-antennary glycans	A4FGE = ((0/4 * (0) + 1/4 * (H4N6F1E1 + H7N6F1E1L2 + H7N6F1E1L3) + 2/4 * (H7N6F1E2L1 + H7N6F1E2L2) + 3/4 * (0) + 4/4 * (0)) / (H4N6F1E1 + H7N6E1L2 + H7N6E2L1 + H7N6F1E1L2 + H7N6F1E2L1 + H7N6E1L3 + H7N6E2L2 + H7N6E3L1 + H7N6F1E1L3 + H7N6F1E2L2)) / ((0/4 * (0) + 1/4 * (H4N6F1E1) + 2/4 * (0) + 3/4 * (0) + 4/4 * (H7N6F1E1L2 + H7N6F1E2L1 + H7N6F1E1L3 + H7N6F1E2L2)) / (H4N6F1E1 + H7N6E1L2 + H7N6E2L1 + H7N6F1E1L2 + H7N6F1E2L1 + H7N6E1L3 + H7N6E2L2 + H7N6E3L1 + H7N6F1E1L3 + H7N6F1E2L2))

Supplementary Table 3. Data quality control. The data quality was checked by measurement of technical replicates of a standard serum sample that were randomly distributed on the plates assessed by calculating the mean, SD (standard deviation), and the relative SD (RSD) for all glycan traits (including directly detected glycan traits and derived glycan traits).

Directly detected glycan traits	Average (Relative abundance)	SD	RSD (mean value is 8.35% for all direct traits)		Derived glycan traits	Average (Relative abundance)	SD	RSD (mean value is 4.06% for all derived traits)
H5N2	0.3836	0.0230	5.99%		TM	0.0203	0.0011	5.46%
H3N3F1	0.0761	0.0210	27.54%		THy	0.0098	0.0007	7.49%
H3N4	0.1905	0.0176	9.24%		TC	0.9686	0.0013	0.13%
H6N2	0.6295	0.0250	3.97%		MHy	2.0621	0.0944	4.58%
H4N3F1	0.0697	0.0079	11.34%		MM	6.9643	0.0497	0.71%
H3N3E1	0.1407	0.0115	8.20%		CA1	0.0070	0.0003	5.03%
H5N3	0.1067	0.0165	15.47%		CA2	0.8374	0.0054	0.64%
H3N4F1	2.2242	0.1430	6.43%		CA3	0.1269	0.0059	4.63%
H4N4	0.3056	0.0120	3.91%		CA4	0.0209	0.0012	5.63%
H3N5	0.1748	0.0237	13.57%		CF	0.3695	0.0078	2.10%
H7N2	0.1841	0.0171	9.30%		CFa	0.0012	0.0001	6.06%
H3N3F1E1	0.0695	0.0084	12.13%		CB	0.1121	0.0024	2.18%
H5N3F1	0.0478	0.0075	15.63%		CG	0.9635	0.0026	0.27%
H4N3E1	0.3670	0.0177	4.82%		CS	0.8587	0.0042	0.49%
H6N3	0.0785	0.0041	5.18%		TA2FS0	12.1743	0.3198	2.63%
H4N4F1	4.4296	0.1428	3.22%		A1F0	0.7494	0.0159	2.12%
H5N4	0.3472	0.0120	3.44%		A2F0	0.6375	0.0054	0.84%
H3N5F1	0.8342	0.0835	10.01%		A3F0	0.5785	0.0114	1.97%
H4N5	0.2138	0.0105	4.91%		A4F0	0.4281	0.1027	23.99%
H5N3L1	0.0427	0.0062	14.42%		A1F	0.2510	0.0133	5.28%
H8N2	0.3525	0.0182	5.18%		A2F	0.3605	0.0061	1.70%

H4N3F1E1	0.1004	0.0089	8.88%		A3F	0.4180	0.0120	2.87%
H4N4L1	0.0392	0.0102	26.07%		A4F	0.5652	0.1022	18.08%
H5N3E1	0.2663	0.0167	6.25%		A3Fa	0.0094	0.0003	3.15%
H5N4F1	2.6586	0.2309	8.69%		A2S0F	0.8972	0.0084	0.94%
H4N4E1	0.4194	0.0147	3.51%		A1L0F	0.2510	0.0133	5.28%
H6N4	0.0269	0.0066	24.57%		A2L0F	0.3548	0.0061	1.71%
H4N5F1	1.4819	0.0389	2.62%		A3L0F	0.2187	0.0045	2.06%
H5N5	0.1690	0.0119	7.07%		A2E0F	0.8693	0.0025	0.29%
H9N2	0.4851	0.0479	9.88%		A3E0F	0.5767	0.0227	3.93%
H5N4L1	0.2649	0.0113	4.27%		A1SF	0.2510	0.0133	5.28%
H6N3E1	0.2210	0.0108	4.89%		A2SF	0.2527	0.0050	1.97%
H4N4F1E1	0.6917	0.0250	3.62%		A3SF	0.4180	0.0120	2.87%
H5N4E1	4.7762	0.1267	2.65%		A4SF	0.5652	0.1022	18.08%
H5N5F1	0.5731	0.0225	3.93%		A2LF	0.4096	0.0169	4.14%
H4N5E1	0.3046	0.0142	4.66%		A3LF	0.5071	0.0202	3.97%
H5N4F1L1	0.5665	0.0097	1.71%		A4LF	0.3820	0.0199	5.22%
H6N4L1	0.0828	0.0067	8.13%		A1EF	0.2510	0.0133	5.28%
H4N7	0.0839	0.0142	16.96%		A2EF	0.2370	0.0050	2.10%
H5N4F1E1	3.3589	0.2284	6.80%		A3EF	0.4147	0.0120	2.89%
H4N5F1E1	0.3828	0.0178	4.65%		A4EF	0.5652	0.1022	18.08%
H5N5E1	1.0621	0.0362	3.41%		A2B	0.1336	0.0032	2.37%
H5N4L2	0.4238	0.0248	5.85%		A2F0B	0.0427	0.0019	4.37%
H5N4E1L1	4.1897	0.1461	3.49%		A2FB	0.2915	0.0062	2.12%
H5N4E2	38.6230	0.5938	1.54%		A2S0B	0.2506	0.0063	2.51%
H5N5F1E1	2.1881	0.0777	3.55%		A2SB	0.1097	0.0026	2.35%
H6N5E1	0.5117	0.0306	5.98%		A2F0S0B	0.3999	0.0135	3.39%
H5N4F1L2	1.0980	0.1301	11.85%		A2F0SB	0.0331	0.0014	4.18%
H4N6F1E1	0.6992	0.1501	21.47%		A2FS0B	0.2344	0.0072	3.08%

H5N4F1E1L1	1.6740	0.0527	3.15%		A2FSB	0.3335	0.0081	2.44%
H4N7E1	0.6067	0.0295	4.86%		A2G	0.9070	0.0020	0.22%
H5N4F1E2	4.0093	0.0615	1.53%		A4G	0.7532	0.0749	9.95%
H6N5F1E1	0.2404	0.0132	5.47%		A2F0G	0.9805	0.0006	0.07%
H5N5E2	0.3038	0.0126	4.15%		A4F0G	0.4281	0.1027	23.99%
H6N5L2	0.0993	0.0071	7.10%		A2FG	0.7759	0.0081	1.04%
H5N5F1E1L1	0.1380	0.0068	4.95%		A4FG	0.3378	0.0069	2.03%
H6N5E1L1	0.5685	0.0433	7.62%		A2S0G	0.5085	0.0216	4.25%
H5N5F1E2	3.0111	0.0562	1.87%		A2SG	0.9863	0.0005	0.05%
H6N5E2	0.5099	0.0298	5.84%		A2F0S0G	0.5544	0.0153	2.76%
H6N5F1L2	0.1325	0.0120	9.04%		A2FS0G	0.5044	0.0204	4.05%
H6N5F1E1L1	0.3050	0.0198	6.50%		A2F0SG	0.9924	0.0005	0.05%
H6N5F1E2	0.3750	0.0288	7.69%		A2FSG	0.9684	0.0008	0.09%
H8N6F2	0.1075	0.0117	10.87%		A2S	0.7451	0.0048	0.64%
H6N5E1L2	0.5549	0.0746	13.44%		A3S	0.9039	0.0021	0.23%
H6N5E2L1	2.9630	0.3386	11.43%		A4S	0.6840	0.0647	9.46%
H6N5E3	1.9973	0.1442	7.22%		A2F0S	0.9097	0.0064	0.71%
H6N5F1E1L2	0.4405	0.0198	4.50%		A3F0S	0.8958	0.0036	0.41%
H6N5F1E2L1	3.2778	0.0601	1.83%		A4F0S	0.3833	0.0926	24.16%
H6N5F1E3	0.2212	0.0185	8.36%		A2FS	0.4600	0.0046	1.00%
H6N5F2E2L1	0.1152	0.0069	5.95%		A3FS	0.9170	0.0051	0.56%
H7N6E1L2	0.1863	0.0310	16.62%		A4FS	0.3128	0.0062	1.98%
H7N6E2L1	0.1716	0.0267	15.57%		A2L	0.0602	0.0005	0.79%
H7N6F1E1L2	0.1181	0.0114	9.67%		A3L	0.2629	0.0043	1.64%
H7N6F1E2L1	0.0845	0.0090	10.69%		A4L	0.3275	0.0562	17.14%
H7N6E1L3	0.1556	0.0314	20.17%		A2F0L	0.0515	0.0016	3.15%
H7N6E2L2	0.2307	0.0479	20.75%		A3F0L	0.2238	0.0079	3.55%

H7N6E3L1	0.1090	0.0112	10.29%		A4F0L	0.1986	0.0500	25.15%
H7N6F1E1L3	0.1250	0.0091	7.24%		A2FL	0.0749	0.0044	5.82%
H7N6F1E2L2	0.1830	0.0087	4.73%		A3FL	0.3171	0.0028	0.87%
					A4FL	0.1286	0.0067	5.18%
					A2E	0.6849	0.0048	0.70%
					A3E	0.6437	0.0044	0.68%
					A4E	0.3586	0.0147	4.10%
					A2F0E	0.8549	0.0056	0.65%
					A3F0E	0.6757	0.0038	0.56%
					A4F0E	0.1847	0.0428	23.17%
					A2FE	0.3867	0.0086	2.23%
					A3FE	0.5994	0.0036	0.59%
					A4FE	0.1732	0.0261	15.08%
					A2GS	0.8219	0.0061	0.74%
					A3GS	0.9039	0.0021	0.23%
					A4GS	0.9091	0.0035	0.38%
					A2F0GS	0.9280	0.0058	0.62%
					A3F0GS	0.8958	0.0036	0.41%
					A4F0GS	0.8947	0.0015	0.17%
					A2FGS	0.5919	0.0072	1.21%
					A3FGS	0.9170	0.0051	0.56%
					A4FGS	0.9261	0.0051	0.55%
					A2GL	0.0665	0.0006	0.96%
					A3GL	0.2629	0.0043	1.64%
					A4GL	0.4311	0.0271	6.28%
					A2F0GL	0.0524	0.0016	3.01%
					A3F0GL	0.2238	0.0079	3.55%
					A4F0GL	0.4635	0.0060	1.29%

					A2FGL	0.0967	0.0044	4.54%
					A3FGL	0.3171	0.0028	0.87%
					A4FGL	0.3904	0.0409	10.49%
					A2GE	0.7554	0.0060	0.79%
					A3GE	0.6437	0.0044	0.68%
					A4GE	0.4819	0.0176	3.65%
					A2F0GE	0.8720	0.0052	0.59%
					A3F0GE	0.6757	0.0038	0.56%
					A4F0GE	0.4308	0.0054	1.25%
					A2FGE	0.4972	0.0122	2.46%
					A3FGE	0.5994	0.0036	0.59%
					A4FGE	0.5388	0.0266	4.94%

Supplementary Table 4. Associations of serum N-glycans with EC (complete list of tests performed). The associations were determined with logistic regression. Age was included as a covariate in the models for the disease-related tests. Odds ratios (OR) are calculated on scaled data. The p-values considered significant with the significance threshold of 4.31E-04 (=0.05/116 derived glycan traits) and indicated in bold in the table. The p-values and OR are reported for the derived glycan traits. HC, healthy controls; EC, endometrial cancer.

Derived glycan traits	beta	SEM	T	p (logistics regression)	OR	p (U test)
TM	0.86	0.32	2.65	8.01E-03	2.36	3.51E-03
**THy**	**-3.51**	**0.86**	**-4.08**	**4.48E-05**	**0.03**	**1.37E-09**
TC	0.24	0.27	0.88	3.78E-01	1.27	3.26E-01
**MHy**	**2.93**	**0.67**	**4.38**	**1.20E-05**	**18.82**	**4.29E-10**
MM	0.41	0.28	1.43	1.51E-01	1.50	8.16E-02
CA1	0.93	0.35	2.68	7.47E-03	2.54	**6.37E-05**
CA2	-0.74	0.31	-2.37	1.76E-02	0.48	6.71E-03
CA3	1.28	0.37	3.41	6.38E-04	3.58	**6.05E-05**
**CA4**	**-1.46**	**0.41**	**-3.59**	**3.32E-04**	**0.23**	**1.41E-05**
**CF**	**-2.50**	**0.60**	**-4.19**	**2.80E-05**	**0.08**	**6.10E-09**
CFa	-0.55	0.29	-1.91	5.63E-02	0.58	1.58E-01
CB	-0.97	0.32	-3.04	2.36E-03	0.38	1.20E-03
CG	0.38	0.27	1.40	1.62E-01	1.47	2.59E-01
CS	1.27	0.37	3.43	6.07E-04	3.57	**2.88E-05**
TA2FS0	-1.27	0.37	-3.46	5.50E-04	0.28	**2.32E-05**
**A1F0**	**1.63**	**0.44**	**3.73**	**1.95E-04**	**5.12**	**2.33E-06**
**A2F0**	**2.12**	**0.51**	**4.12**	**3.72E-05**	**8.33**	**6.78E-08**
A3F0	0.62	0.29	2.11	3.45E-02	1.86	4.43E-02
A4F0	9.76	4.19	2.33	1.99E-02	>100	**2.09E-12**
**A1F**	**-1.63**	**0.44**	**-3.73**	**1.94E-04**	**0.20**	**2.33E-06**
**A2F**	**-2.12**	**0.51**	**-4.12**	**3.73E-05**	**0.12**	**6.78E-08**
A3F	-0.62	0.29	-2.12	3.43E-02	0.54	4.43E-02
A4F	-9.79	4.20	-2.33	1.98E-02	0.00	**2.09E-12**

A3Fa	-1.02	0.35	-2.94	3.25E-03	0.36	1.11E-02
A2S0F	-0.83	0.33	-2.52	1.18E-02	0.43	2.76E-03
**A1L0F**	**-1.63**	**0.44**	**-3.73**	**1.94E-04**	**0.20**	**2.33E-06**
**A2L0F**	**-2.15**	**0.52**	**-4.11**	**4.02E-05**	**0.12**	**6.78E-08**
**A3L0F**	**-2.47**	**0.59**	**-4.22**	**2.44E-05**	**0.08**	**1.36E-08**
A2E0F	-0.06	0.25	-0.23	8.21E-01	0.95	9.51E-01
**A3E0F**	**-1.84**	**0.46**	**-4.01**	**6.06E-05**	**0.16**	**4.63E-07**
**A1SF**	**-1.63**	**0.44**	**-3.73**	**1.94E-04**	**0.20**	**2.33E-06**
**A2SF**	**-2.08**	**0.50**	**-4.13**	**3.65E-05**	**0.12**	**7.77E-08**
A3SF	-0.62	0.29	-2.12	3.43E-02	0.54	4.43E-02
A4SF	-9.79	4.20	-2.33	1.98E-02	0.00	**2.09E-12**
A2LF	-0.79	0.32	-2.49	1.29E-02	0.46	4.69E-02
A3LF	-0.39	0.28	-1.43	1.53E-01	0.67	3.15E-01
A4LF	-0.25	0.26	-0.95	3.44E-01	0.78	4.54E-01
**A1EF**	**-1.63**	**0.44**	**-3.73**	**1.94E-04**	**0.20**	**2.33E-06**
**A2EF**	**-2.01**	**0.49**	**-4.10**	**4.06E-05**	**0.13**	**1.09E-07**
A3EF	-0.51	0.28	-1.79	7.35E-02	0.60	1.38E-01
A4EF	-9.79	4.20	-2.33	1.98E-02	0.00	**2.09E-12**
A2B	-0.91	0.31	-2.95	3.17E-03	0.40	1.69E-03
A2F0B	0.36	0.29	1.23	2.20E-01	1.43	3.51E-01
A2FB	0.03	0.26	0.11	9.14E-01	1.03	4.77E-01
A2S0B	0.29	0.27	1.08	2.78E-01	1.34	1.00E-01
A2SB	-0.83	0.30	-2.74	6.14E-03	0.43	3.24E-03
A2F0S0B	1.02	0.38	2.67	7.68E-03	2.78	2.00E-03
A2F0SB	0.40	0.31	1.31	1.89E-01	1.50	3.45E-01
A2FS0B	0.02	0.26	0.09	9.31E-01	1.02	4.40E-01
A2FSB	0.02	0.25	0.09	9.29E-01	1.02	5.81E-01

A2G	0.78	0.30	2.58	1.00E-02	2.19	9.31E-03
A4G	265.34	80261.81	0.00	9.97E-01	>100	**1.75E-12**
A2F0G	1.57	0.46	3.40	6.82E-04	4.79	**3.39E-05**
A4F0G	9.76	4.19	2.33	1.99E-02	>100	**2.09E-12**
A2FG	-0.04	0.26	-0.16	8.75E-01	0.96	4.54E-01
A4FG	-0.29	0.28	-1.05	2.92E-01	0.75	3.82E-02
A2S0G	-0.45	0.31	-1.47	1.41E-01	0.64	3.38E-02
**A2SG**	**3.98**	**0.97**	**4.09**	**4.34E-05**	**53.34**	**6.79E-11**
A2F0S0G	-0.13	0.28	-0.47	6.37E-01	0.88	2.80E-01
A2FS0G	-0.51	0.31	-1.63	1.04E-01	0.60	1.98E-02
**A2F0SG**	**4.39**	**1.15**	**3.81**	**1.42E-04**	**81.02**	**3.13E-10**
A2FSG	0.87	0.30	2.87	4.17E-03	2.38	1.15E-03
**A2S**	**1.66**	**0.44**	**3.74**	**1.84E-04**	**5.28**	**1.62E-06**
**A3S**	**2.77**	**0.69**	**4.00**	**6.23E-05**	**15.97**	**1.68E-08**
A4S	259.70	79698.04	0.00	9.97E-01	>100	**1.92E-12**
A2F0S	0.40	0.26	1.50	1.32E-01	1.49	4.05E-02
**A3F0S**	**1.64**	**0.46**	**3.57**	**3.56E-04**	**5.17**	**2.73E-05**
A4F0S	9.60	4.10	2.34	1.92E-02	>100	**2.49E-12**
A2FS	0.49	0.28	1.72	8.46E-02	1.63	7.53E-02
A3FS	1.58	0.50	3.19	1.41E-03	4.88	**7.44E-05**
A4FS	-0.49	0.30	-1.66	9.69E-02	0.61	8.99E-03
A2L	-0.85	0.31	-2.77	5.54E-03	0.43	1.10E-03
A3L	-0.66	0.30	-2.24	2.54E-02	0.51	2.88E-03
A4L	624.88	101446.81	0.01	9.95E-01	>100	**1.92E-12**
**A2F0L**	**-2.15**	**0.54**	**-3.95**	**7.83E-05**	**0.12**	**6.33E-08**
A3F0L	-0.31	0.28	-1.12	2.63E-01	0.73	4.69E-02
A4F0L	8.63	3.42	2.53	1.15E-02	>100	**5.00E-12**
A2FL	0.54	0.29	1.87	6.21E-02	1.72	2.11E-02

A3FL	-0.22	0.26	-0.84	4.03E-01	0.81	3.64E-01
**A4FL**	**4.08**	**1.06**	**3.86**	**1.14E-04**	**59.22**	**1.72E-09**
**A2E**	**2.07**	**0.52**	**3.96**	**7.38E-05**	**7.91**	**1.17E-07**
**A3E**	**3.46**	**0.91**	**3.81**	**1.39E-04**	**31.67**	**3.38E-09**
A4E	17.75	11.87	1.49	1.35E-01	>100	**1.61E-12**
A2F0E	1.00	0.32	3.08	2.05E-03	2.71	**2.34E-04**
A3F0E	1.72	0.49	3.49	4.77E-04	5.59	**8.72E-07**
A4F0E	7.96	2.89	2.75	5.88E-03	>100	**2.09E-12**
A2FE	0.41	0.27	1.51	1.32E-01	1.51	1.19E-01
**A3FE**	**3.46**	**0.92**	**3.74**	**1.87E-04**	**31.66**	**7.07E-09**
**A4FE**	**-3.82**	**0.95**	**-4.04**	**5.40E-05**	**0.02**	**1.11E-10**
**A2GS**	**2.01**	**0.54**	**3.73**	**1.88E-04**	**7.46**	**9.28E-07**
**A3GS**	**2.77**	**0.69**	**4.00**	**6.23E-05**	**15.97**	**1.68E-08**
**A4GS**	**-4.50**	**1.19**	**-3.80**	**1.45E-04**	**0.01**	**9.94E-12**
A2F0GS	0.10	0.25	0.40	6.91E-01	1.10	2.39E-01
**A3F0GS**	**1.64**	**0.46**	**3.57**	**3.56E-04**	**5.17**	**2.73E-05**
A4F0GS	0.12	0.26	0.48	6.32E-01	1.13	9.80E-01
A2FGS	0.92	0.36	2.58	9.98E-03	2.51	1.20E-03
A3FGS	1.58	0.50	3.19	1.41E-03	4.88	**7.44E-05**
**A4FGS**	**-4.74**	**1.24**	**-3.83**	**1.27E-04**	**0.01**	**8.67E-11**
A2GL	-1.23	0.36	-3.43	6.09E-04	0.29	**5.17E-05**
A3GL	-0.66	0.30	-2.24	2.54E-02	0.51	2.88E-03
A4GL	10.36	4.31	2.41	1.61E-02	>100	**5.94E-12**
**A2F0GL**	**-2.22**	**0.56**	**-3.95**	**7.69E-05**	**0.11**	**5.52E-08**
A3F0GL	-0.31	0.28	-1.12	2.63E-01	0.73	4.69E-02
A4F0GL	-0.11	0.27	-0.43	6.69E-01	0.89	3.15E-01
A2FGL	0.54	0.29	1.85	6.48E-02	1.72	1.23E-02

A3FGL	-0.22	0.26	-0.84	4.03E-01	0.81	3.64E-01
A4FGL	9.43	4.01	2.35	1.88E-02	>100	**5.45E-12**
**A2GE**	**2.98**	**0.77**	**3.86**	**1.13E-04**	**19.66**	**3.91E-08**
**A3GE**	**3.46**	**0.91**	**3.81**	**1.39E-04**	**31.67**	**3.38E-09**
A4GE	-1779.02	152685.85	-0.01	9.91E-01	0.00	**1.92E-12**
A2F0GE	0.82	0.30	2.73	6.43E-03	2.27	1.37E-03
A3F0GE	1.72	0.49	3.49	4.77E-04	5.59	**8.72E-07**
A4F0GE	0.18	0.26	0.70	4.84E-01	1.20	2.29E-01
A2FGE	0.74	0.32	2.32	2.02E-02	2.09	2.55E-03
**A3FGE**	**3.46**	**0.92**	**3.74**	**1.87E-04**	**31.66**	**7.07E-09**
A4FGE	-10.05	4.68	-2.15	3.16E-02	0.00	**5.45E-12**

Supplementary Table 5. The diagnostic value of the optimized glycan panel built with the four most important (discriminative and biologically reliable) derived glycan traits using different models/algorithms.

Model/Algorithm (used in the multivariate ROC exploratory analyses)	AUC	95%CI	Average accuracy
Random forest	0.993	0.955-1	0.959
Linear SVM	0.987	0.946-1	0.940
PLS	0.989	0.950-1	0.957

Supplementary Table 6. Associations of serum N-glycan traits and classical gynecologic tumor markers with the differentiation type (well or poorly differentiated) of EC. The associations were determined with logistic regression. Age was included as a covariate in the models. Odds ratios (OR) are calculated on scaled data.

Derived glycan traits or classical gynecologic tumor markers	beta	SEM	T	p (logistics regression)	OR
**Derived glycan traits**					
THy	-2.24	0.95	-2.36	**0.018**	0.11
TC	1.58	0.75	2.10	**0.035**	4.86
**Classical gynecologic tumor markers**					
CA125	0.82	0.63	1.30	0.194	/
CA199	-0.67	0.93	-0.73	0.468	/
CEA	-0.14	0.63	-0.22	0.828	/
